# Measurement of Immunoglobulin Intraclonal diversification refines the clinical impact of IGHV mutational status in chronic lymphocytic leukemia

**DOI:** 10.1038/s41375-025-02650-2

**Published:** 2025-06-18

**Authors:** Filippo Vit, Tamara Bittolo, Antonella Zucchetto, Robel Papotti, Erika Tissino, Federico Pozzo, Annalisa Gaglio, Andrea Stacchetti, Eva Zaina, Ilaria Cattarossi, Paola Varaschin, Paola Nanni, Michele Berton, Alessandra Braida, Francesca Maria Rossi, Massimo Degan, Jerry Polesel, Roberta Laureana, Annalisa Chiarenza, Jacopo Olivieri, Annalisa Biagi, Giovanni D’Arena, Marco Rossi, Luca Laurenti, Agostino Tafuri, Pietro Bulian, Alberto Zamò, Ellen Leich, Andreas Rosenwald, Evgeny Arons, Robert Kreitman, Massimo Gentile, Massimiliano Postorino, Francesco Zaja, Francesco Di Raimondo, Maria Ilaria Del Principe, Valter Gattei, Riccardo Bomben

**Affiliations:** 1 Clinical and Experimental Onco-Haematology Unit, Centro di Riferimento Oncologico di Aviano (CRO), IRCCS, Aviano, Italy; 2 Unit of Cancer Epidemiology, Centro di Riferimento Oncologico di Aviano (CRO) IRCCS, Aviano, Italy; 3 Division of Hematology, University of Tor Vergata, Rome, Italy; 4 Division of Haematology, Ferrarotto Hospital, Catania, Italy; 5 Clinica Ematologica, Centro Trapianti e Terapie Cellulari “Carlo Melzi” DISM, Azienda Ospedaliera Universitaria S. Maria Misericordia, Udine, Italy; 6 Hematology and Transplant Unit, Ospedale S.M.Goretti, AUSL Latina, Latina, Italy; 7 Haematology Unit, Presidio Ospedaliero S. Luca, ASL Salerno, Salerno, Italy; 8 Department of Hematology-Oncology, Dulbecco University Hospital, Catanzaro, Italy; 9 Fondazione Universitaria Policlinico A Gemelli di Roma, Roma, Italy; 10 Department of Clinical and Molecular Medicine and Hematology, Sant’Andrea - University Hospital - Sapienza, University of Rome, Rome, Italy; 11 Institute of Pathology, University of Würzburg, Würzburg, Germany; 12 Center for Cancer Research, National Cancer Institute, Bethesda, MD USA; 13 Hematology Unit, Azienda Ospedaliera Annunziata, Cosenza, Italy; 14 Department of Pharmacy, Health and Nutritional Science, University of Calabria, Rende, Italy; 15 Department of Medical, Surgical and Health Sciences, University of Trieste, Trieste, Italy

**Keywords:** Cancer genomics, Chronic lymphocytic leukaemia

## Abstract

Chronic lymphocytic leukemia (CLL) cells may bear mutations in IGHV genes, the 2%-cutoff allowing to discriminate two subsets, unmutated (U)- or mutated (M)-CLL, with different clinical course. IGHV genes may also incorporate additional ongoing mutations, a phenomenon known as intraclonal diversification (ID). Here, through an original bioinformatic workflow for NGS data, we used the inverse Simpson Index (iSI) as diversity measure among IGHV sequences to dichotomize cases with different ID levels into ID_high_ (iSI ≥ 1.2) vs. ID_low_ (iSI < 1.2) both in CLL (*n* = 983) and in other lymphoproliferative disorders (LPD; *n* = 127). In CLL, ID_high_ cases accounted for 14.6%, overrepresented in M-CLL (*P* = *0.0028*), while higher percentages were documented in GC-derived LPD. In M-CLL (*n* = 396), ID_high_ patients (*n* = 69) experienced longer time-to-first treatment than ID_low_ patients (*P* = 0.015), and multivariate analyses (*n *= 299) confirmed ID as independent variable. IGHV gene mutations of ID_high_ cases had molecular signatures indicating ongoing activity of the AID)/Polη-dependent machinery; consistently, ID_high_ M-CLL expressed higher levels of AID transcripts than ID_low_ M-CLL (*P* = 0.012). In conclusion, we propose a robust NGS protocol to quantitatively evaluate ID in CLL, demonstrating that: i) all CLL patients presented ID although at various degree; ii) high degree of ID has clinical relevance identifying a M-CLL subset with better outcome.

## Introduction

In chronic lymphocytic leukemia (CLL), a neoplastic disease characterized by highly variable clinical courses [1–4], the mutational status of the heavy chain variable region of the immunoglobulin (IGHV) genes represents one of the most relevant prognostic/predictive biomarkers [5]. In particular, CLL expressing a mutational load of IGHV genes lower than or equal to the established cut-off of 2% are identified as unmutated (U-CLL), and are associated with a poorer prognosis compared to CLL carrying IGHV gene mutations exceeding the 2% cut-off (mutated CLL, M-CLL) [3]. Nowadays, such a dichotomized IGHV mutational status is included in several prognostic algorithms predicting the time-to-first treatment (TTFT) [2, 4], as well as the response to chemo-immunotherapy regimens (CLL-IPI) [5, 6]. Conversely, in the context of novel target therapies the clinical impact of the IGHV gene mutational status is less clear and still to be evaluated [1–3, 7–9].

Despite the dichotomous categorization according to the IGHV gene mutational status, CLL cases may exhibit marks of ongoing mutations in the context of IGHV genes, a phenomenon known as intraclonal diversification (ID). After the neoplastic transformation of mature B cells, CLL may incorporate novel mutations in IGHV sequences due to the maintenance of the physiologic process of somatic hypermutation (SHM) physiologically responsible for the refinement of IG affinity and the generation of heterogeneity of the IG repertoire [10]. In this regard, Gurrieri et al. demonstrated that the physiological SHM machinery, driven by the activated-induced cytidine deaminase (AID), is frequently activated in CLL and responsible for the ID phenomenon [11, 12]. Similarly, Degan et al. confirmed that these IGHV ongoing mutations found in CLL bore the signature of AID, and were compatible with reparation mechanisms involving both AID and error-prone bypass lesion DNA polymerases [10, 13]. These studies, however, since performed using Sanger sequencing, had limited capacity with regard to analytical depth and breadth.

The advent of the high-throughput Next-Generation Sequencing (NGS) revolutionized the study of the immunological repertoire due to an increased discrimination power [14]. Despite the use of NGS allows a finer discrimination and quantification of IG gene repertoire, both at the clonal and the subclonal levels [14], the NGS technique by itself may retain artifact/bias in part overcome by the introduction of Unique Molecular Identifiers (UMI) for the generation of the Repertoire Sequencing (RepSeq) library [15, 16]. Following this reasoning, Bagnara et al. [17], by applying an UMI-based multiplex amplification protocol to study ID in CLL, could confirm previous findings, without demonstrating correlation between ID and clinically relevant parameters [11, 13, 17]. In addition, other studies limited ID analyses to specific CLL stereotyped subsets [18, 19].

More recently, a bioinformatic tool has been presented characterizing the mutations occurring in the context of the ID process and their interconnections, with the aim to build “mutational pathways” [18, 20]. However, so far, the low number of tested samples due to the complexity of a UMI-based approach, and the scarcity of ad-hoc bioinformatic packages have hampered a high-throughput characterization of ID in CLL providing clinical correlations.

In this study, we evaluated ID in a large cohort of CLL and other germinal center (GC)- and non-GC-derived lymphoproliferative disorders (LPD) by applying a deep NGS strategy for IG repertoire analyzed through an ad-hoc developed UMI-independent bioinformatics pipeline. By taking advantage of this original approach, we have been able to provide compelling evidence that ID is a phenomenon present in CLL, although rarer than in other GC-derived LPD, and more frequent in M- than U-CLL. In particular, M-CLL with substantial ID (ID_high_ M-CLL) were clinically associated with longer TTFT than M-CLL without substantial ID (ID_low_ M-CLL).

## Material and methods

### CLL and LPD cohorts

**Fig. 1:**
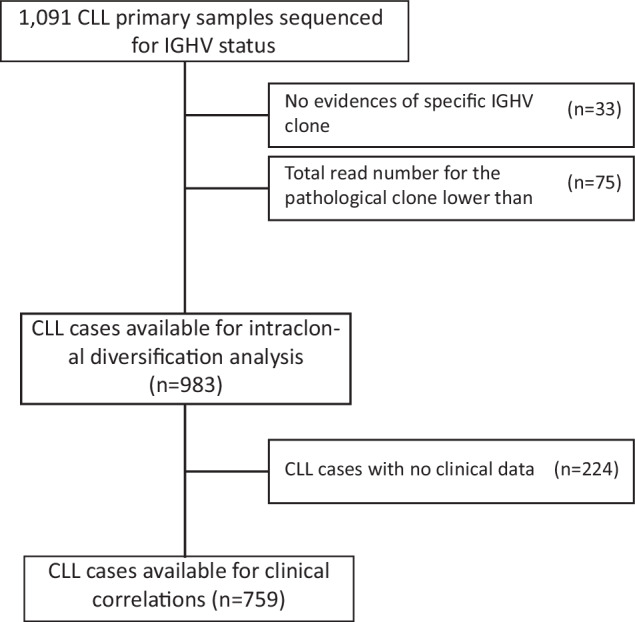
Flow-chart of the study with the number of patients analyzed.

The study comprises a multicenter retrospective cohort of 1091 CLL primary samples diagnosed from 2005 to 2021 and referred to a single institution (Clinical and Experimental Onco-Hematology Unit, Centro di Riferimento Oncologico, I.R.C.C.S., Aviano, Italy) for molecular and cytogenetic analyses (Fig. 1). IGHV sequencing data were directly retrieved from the NGS analyses performed in the context of diagnostic procedures for cases received by the reference center after 2015; cases diagnosed before 2015, whose IGHV sequencing were originally done by Sanger, were re-tested by NGS from archived nucleic acids (Table S1). All analyses were performed on a sample received at the time of diagnosis/first presentation, always before therapy. All patients were diagnosed and treated according to iwCLL guidelines [1, 2]. Among 1091 patients, TTFT was available for 759 CLL patients (Fig. 1), updated as of August 2023. Out of 759 cases, 320 were treated with a median TTFT from CLL diagnosis of 29 months (95% CI 26.0–31.0, range 0–199). Analysis of clonal evolution of ID was performed on 33 patients with sequential time-points available (median number of time-points available = 2, range 2–5) and with a median time interval between sequential samples of 25.8 months (range 0.7–112.8 months). For the comparison of ID levels, cohorts of LPD encompassing different stages of mature B cell differentiation, including 45 mantle cell lymphoma (MCL), 28 diffuse large B cell lymphoma (DLBCL), 40 follicular lymphoma (FL), 14 hairy cell leukemia (HCL) cases were collected. The study was carried out in accordance with the declaration of Helsinki upon IRB approval (Approval n. IRB-05-2010 and n. IRB-05- 2015, Centro di Riferimento Oncologico of Aviano; Approval n. 10C0066, National Cancer Institute; Approval, University of Würzburg, January 17th, 2006), and informed consent.

### IGHV sequencing and library preparation with UMI-tagged IGHV

DNA/RNA extraction, RNA retro-transcription, IGHV amplification and NGS sequencing were performed according to standard protocols (details in Supplemental Materials and Methods). The major IG stereotyped CLL subsets were assigned with ARResT Tool (https://bat.infspire.org/arrest/assignsubsets/) [21].

UMI-tagged libraries were generated for evaluation of the immunoglobulin repertoire, according to the protocol from Khan et. al. by utilizing UMI generated as 3 non-randomic sequences interspersed by 3 bp spacers (details in Supplemental Materials and Methods, Figure S1 and Table S2) [15].

### Hotspot and coldspot mutability calculation

To evaluate the mutability within AID-specific hotspots/coldspots [12, 22, 23], we applied the igphyml algorythm on corrected data [24]. To confirm the results, we applied a custom python script which retrieves the number of mutations per sample and catalogues them as compatible with SHM signature or not (details in Supplemental Materials and Methods).

### *AID* mRNA quantification with qPCR

*AID* and *Beta-2-Microglobulin* (β2 M) mRNA levels, the latter as control gene, were assessed through Taqman-qPCR assay (Thermo Fisher) using a CFX96 PCR System (BioRad). The relative expression was calculated with the ∆∆CT method using MEC1 cell line as a positive control.

### Other CLL characterizations

Interphase FISH was performed to detect del17p, 11q22.3 deletion (del11q), 13q14 deletion (del13p), and trisomy 12 (tris12) [25, 26]. CLL patients were also characterized for age, sex, Rai/Binet staging, CD38 and CD49d expression, as well as by a standard immunophenotypic profile which included evaluation of the expression of CD5, CD23, CD20, FMC7, CD43, CD79b, SmIg, IgM and IgD, as previously reported [27, 28].

### Survival analysis

All the statistical analyses were performed with R programming language. TTFT was computed from the date of diagnosis to the date of first treatment (events) or last follow-up (censoring). Molecular studies were blinded to the study end points. To compare differences in TTFT we applied the Log-rank tests, and Kaplan Meier curves were used for visualization [29]. ID levels, as evaluated according to the iSI, were tested for possible cut-off values of prognostic relevance with maximally selected log-rank statistics [30]. Cox models were used to verify independent prognostic power of parameters; model minimization was performed by stepwise backward elimination. Departure from proportionality in hazard was tested in all Cox models. Internal bootstrapping validations were as reported [31], by performing at least 500 replications. In all comparisons a P value level of 0.05 was established as statistically significant.

## Results

### Measurement of ID in CLL by a UMI-independent strategy

Out of 1091 CLL primary samples, an identifiable pathological clone was retrieved in 1058 CLL samples (97.0%) either by IGHV Leader or FR1 assays, while in 33 cases a prevalent CLL clone was not detected (Fig. 1 and Figure S2). Among these 1058 cases, 75 were excluded due to <5000 total number of reads referring to the pathological clone, to reach the final number of 983 CLL evaluable samples (Fig. 1 and Figure S2A). Accordingly, the median number of sequences analyzed in the context of the final cohort is 15.407 (range 5.192–70.078; interquartile range 25%–75%; 8.525–21.480; Figure S2B)

Preliminary to ID determination, Fastq files were analyzed using a custom bioinformatic pipeline which includes an original custom python script to identify and correct systematic sequencing errors which may affect ID evaluation (Italian Patent 102022000027138/publication #IT202200027138A1), as detailed in Supplemental Materials and Methods and Figure S3–S6 [32–35].

**Fig. 2:**
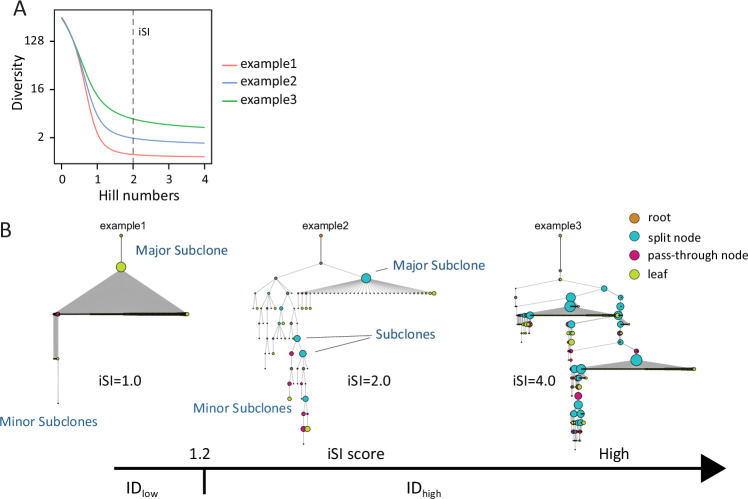
Illustrative phylogenetic trees in dependence of presence of IGHV Intraclonal Diversification (ID). **A** ID was calculated from the Hill number-based diversity profiles of diversity indices. Among the different Hill number-derived indices, the inverse Simpson index (iSI, corresponding to a Hill number = 2) was selected. **B** Phylogenetic tree examples of three different CLL samples with increasing iSI. Nodes in the tree can be either the root node (orange node), leaves (sequences of cells that had no descendants; green nodes), or internal nodes. Internal nodes can be either split nodes, those with more than one child (light blue nodes); or pass-through nodes, those with exactly one child (red nodes). Size of the circle corresponds to the percentage of the specific subclone inside the pathological clone.

After correction of systematic sequencing errors, as hallmark of ID, a subclone was defined as having the same IGHV, IGHD, IGHJ, and CDR3 sequence of the main clone but differing from the main clone by at least one nucleotide somewhere in the sequence. To measure ID inside the CLL clone, we took advantage from rarefaction curves extrapolated through Hill number-based diversity profiles of diversity indices; in this regard, Fig. 2A reports three different rarefaction curves calculated according to the generalized form of Hill’s numbers [36]. In particular, among the different Hill number-derived indices, we used the inverse Simpson index (iSI, corresponding to a Hill number = 2), as an index that take into consideration the proportional abundances of the different subclones within the immunoglobulin population (Fig. 2B) [36, 37].

For iSI calculation, all the sequences with a frequency ≥0.1% of the total number of reads belonging to that clone were considered. Only cases with a total read number ≥5000 were considered for iSI calculation (Figure S2) to avoid iSI overestimation due to low count clones as well as to have the possibility to identify subclones with 0.1% frequency (at least 5 supporting reads per subclone).

**Fig. 3:**
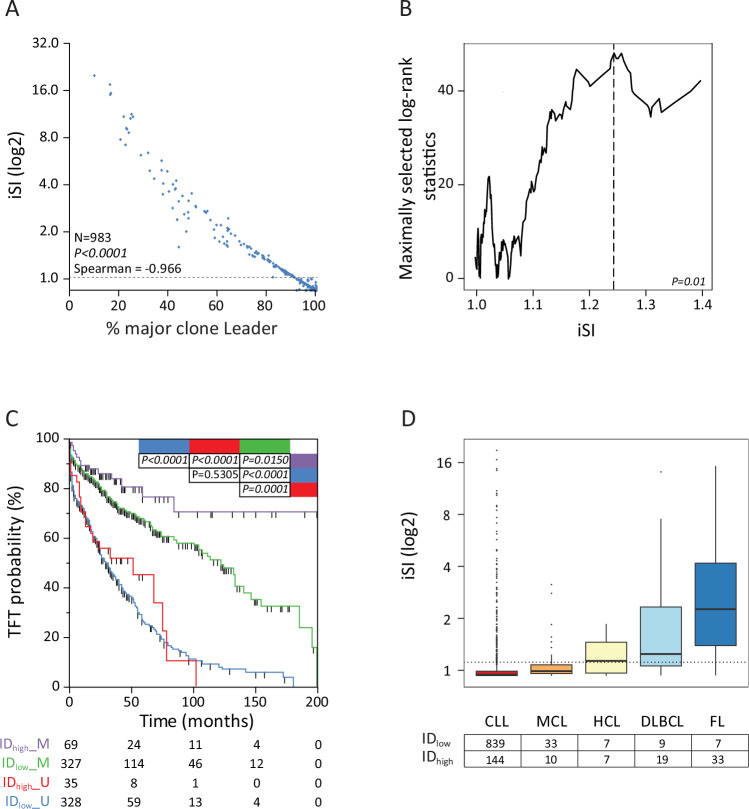
Diversity score in lymphoproliferative disease. **A** The scatter plot depicts the iSI calculated for 983 CLL versus the percentage of the Major Clone (clone with the higher percentage) inside the identified pathological clone. **B** Maximally selected rank statistics graphs for the determination of the best iSI cutoff. **C** Kaplan-Meier curves comparing TTFT probabilities of 327 M IGHV cases with low intraclonal (iSI < 1.2; ID_low_; green line), 69 M IGHV cases with high intraclonal (iSI ≥ 1.2; ID_high_) (purple line), 328 U IGHV cases with low intraclonal (light blue line), and 35 U IGHV cases with high intraclonal (red line). The number of patients in each group is reported; *P* value refers to log-rank test. **D** Boxplots report the distribution of diversity score calculated by means of Inverse Simpson Index (iSI) in different lymphoproliferative disease. Dotted line refers to iSI cutoff of 1.2.

When plotting the iSI values against the percentage of the major subclone inside the pathological clone in the 983 CLL evaluable samples, a continuum of iSI values was observed (median iSI = 1.016, range 1.0–20.4) inversely correlating (Spearman’s coefficient = −0.966; *P* < *0.0001*) with the percentage of the major subclone (Fig. 3A).

No quantitative differences in term of major/minor subclone composition and iSI were found by comparing 91 cases in which ID was investigated by using IGHV Leader-specific primers starting from cDNA and IGHV FR1-specific primers starting from DNA when our custom pipeline of analysis was employed (Figure S7A–C), suggesting that RNA transcription and/or translation should not be involved in the generation of ID.

### Comparison between UMI-independent and UMI-dependent strategies in ID measurement in CLL

Fifty-two cases, previously analyzed with the IGHV Leader assay, were subjected to an UMI strategies to investigate whether our UMI-independent strategy could be prone to possible random PCR artifacts [15, 16].

In this context, fastq were merged with vsearch and UMI were extracted from fastq with a custom python script (Figure S8). In addition, again to correct possible systematic errors (Figure S4), not bypassed by UMI usage for IGHV sequencing, the same original custom python-based bioinformatic pipeline (Figure S3), with minor changes, was employed (Figure S8). Again, iSI was calculated for ID assessment as reported above (see Figure S3 and Supplemental Materials and Methods).

The frequency distribution of the major and of the minor pathological subclones, as identified by both assays, showed a significantly high correlation (*R*^2^ = 0.9467 and *R*^2^ = 0.8503, respectively; *P* < *0*.0001; Figure S7D-E). Consistently, a significant correlation was also obtained by comparing the iSI scores between samples processed with or without a UMI-based protocol (*R*^2^ = 0.8945; *P* < *0.0001*; Figure S7F). Overall, this demonstrated that our UMI-independent approach is able to recapitulate UMI-generated results in terms of sequence complexity and ID.

### Clinical relevance of ID measurement in CLL

To test whether ID measurement may be a clinically relevant parameter in CLL, we retrieved the TTFT of 759 patients (Fig. 1). We firstly confirmed that M-CLL patients had longer TTFT than U-CLL patients (median TTFT: 132.0 *versus* 31.0 months; *P* < *0.0001*, Figure S9).

Then, we sought for the optimal cut-off point yielding the best separation of CLL with or without significant ID levels into two subgroups with different TTFT. According to the trend of standardized log-rank statistics plotted along with iSI values as measures of ID, the optimal cut-off point (Fig. 3B) was chosen at an iSI value set at 1.23 (rounded at 1.2 for our purposes). According to this value, the cohort of 759 cases with clinical data available was split into CLL cases with substantial ID (ID_high_, iSI > 1.2, *n* = 104) and cases with lower level or no evidence of ID (ID_low_, iSI ≤ 1.2, *n* = 655).

The clinical impact of ID in CLL was defined by introducing ID into a conventional M/U-CLL prognostic stratification. As shown in Fig. 3C, M-CLL patients with ID_high_ (*n* = 69) witnessed significantly longer TTFT respect to their ID_low_ counterpart (*n* = 327; median TTFT not reached *versus* 122.0 months, respectively; *P* = *0.015*). Conversely, no significant variation in TTFT was observed comparing ID_high_ (*n* = 35) and ID_low_ U-CLL (*n* = 328; median TTFT 51.0 months *versus* 31.0 months, respectively; *P* = 0.5305, Fig. 3C).

In the context of M-CLL (*n* = 299), ID remained independent TTFT predictor (*P* = *0.022*) after adjusting for possible confounders, including Rai staging and other biological factors (CD49d, and CD38 expression, and genomic abnormalities; Table 1). Consistently, independent variables were the most frequently selected by internal bootstrap validation (Table 1).

**Table 1:** Univariable and multivariable analyses of TTFT (*n* = 299).

	UVA				MVA^a^				Bootstrap (1000 replications)			
	HR	LCI	UCI	*P*	HR	LCI	UCI	*P*	HR	LCI	UCI	% *P* < 0.05
*Gender (Male)*	1.19	0.79	1.78	0.4045	–							
*Age (≥65 y)*	1.07	0.72	1.59	0.7524	–							
*Rai stage (II-III-IV)*	8.57	5.61	13.10	***<0.0001***	7.97	5.06	12.57	***<0.0001***	8.83	5.76	15.08	100%
*CD49d (high)*	2.35	1.59	3.48	***<0.0001***	1.85	1.16	2.93	***0.0092***	1.86	1.05	2.95	67.4%
*CD38 (high)*	1.62	1.07	2.46	***0.0237***	ni							
*Intraclonal diversification (ID*_*low*_*_M)*	2.01	1.07	3.77	***0.0293***	2.11	1.11	4.02	***0.0224***	2.20	1.28	4.38	67.8%
*Genetic model (reference: normal)*												
* del13q*	0.88	0.52	1.51	0.6466	1.00	0.57	1.76	0.9880	0.97	0.61	1.57	4.0%
* tris12*	1.96	1.06	3.61	***0.0317***	1.03	0.54	1.95	0.9351	1.05	0.55	2.21	10.6%
* del11q and/or del17p*^b^	3.92	2.15	7.14	***<0.0001***	2.38	1.27	4.47	***0.0070***	2.51	1.43	4.90	78.0%

CD49d low vs high according to 30% cutoff; CD38 low vs high according to 20% cutoff; Dohner classification according to FISH analysis of del17p, del11q, tris12, and del 13q; M_C, IGHV mutated (M), <98% identity with germ line and absence of intraclonal diversification; M_I, IGHV M, and presence of intraclonal diversification.

*P*-values less than 0.05 are reported in bold.

*TTFT* time to first treatment, *UVA* univariable analysis, *MVA* multivariable analysis, *HR* Hazard Ratio, *CI* confidence interval, *LCI* 95% lower CI, *UCI* 95% Upper CI; -: not used in the final model, *ni* not included in the final model.

^a^Multivariable analysis was carried out using the following variables (*n* = 299): Rai stage, CD49d, CD38, hierarchical genetic model (del17p and/or del11q with or wothout tris12 and/or del13q; tris12 with or without del13q; del13q), intraclonal diversification.

^b^Treated as categorical variables respect to normal (absence of del17p, del11q, tris12, and del13q) cases.

### Biological validation of iSI cut-off in other LPD

To further validate our approach for ID determination and the 1.2 iSI cutoff, ID was investigated in other B-cell malignancies better characterized in the literature regarding ID. As reported in Fig. 3D, the median iSI for HC, DLBCL and FL was 1.22 (range: 1.0–2.0), 1.34 (range 1.0–15.3) and 2.44 (range 1.0–16.56), respectively. By applying the same iSI cutoff found in CLL, 50% of HCL (7/14), 73.0% of DLBCL (19/26) and 82.5% of FL samples (33/40) were classified ID_high_ in keeping with literature data [38–42]. On the contrary, 43 MCL cases, usually lacking ID features [43], presented a median iSI of 1.1 (range: 1.0–3.4), with only 23.2% of cases (10/43) turning out ID_high_ (Fig. 3D).

### Comparison of ID measurement by iSI with the intraclonal complexity determined by the immunoglobulin phylogenetic tree

Bioinformatics tools have been presented for the characterization of mutations occurring in the context of the ID process and their interconnections, with the aim to build “mutational pathways” of different complexity [18, 20]. As reported in Figure S10, ID_high_ CLL, identified according to our approach, always showed the highest levels of the different parameters of the phylogenetic trees which were associated with a higher intraclonal complexity of immunoglobulin [18, 20].

### Characterization of CLL with different ID levels: gene usage and IG stereotyped subsets

**Fig. 4:**
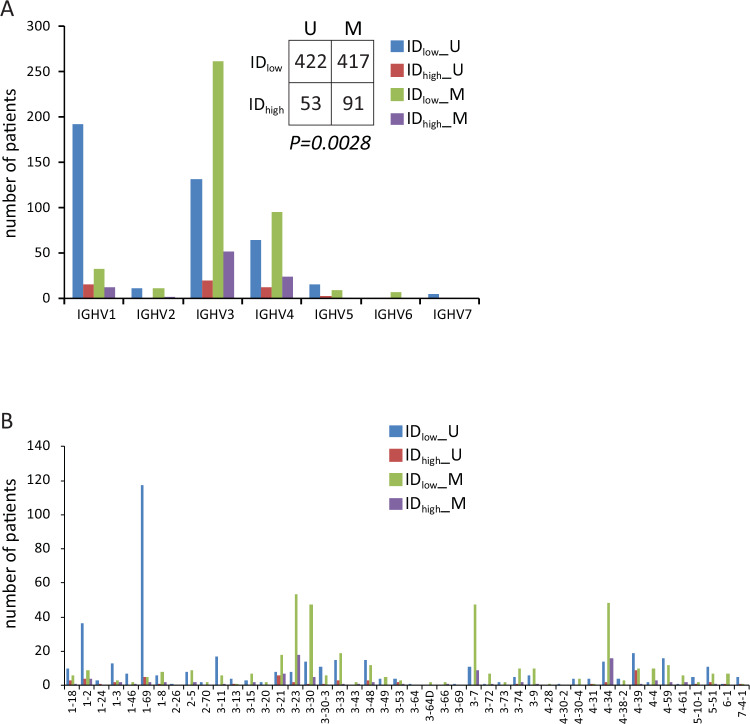
Distribution of IGHV families and genes in the CLL cohort among samples with low or high intraclonal diversification (ID). **A** The barchart reports the number of CLL cases in dependence of IGHV families according the IGHV mutational status and divided by the presence or not of ID. **B** The barchart reports the number of CLL cases in dependence of IGHV genes divided according to the IGHV mutational status (mutated: M, unmutated: U) and the presence or not of ID. Light-blue bars represent unmutated IGHV (U-CLL) and ID_low_ cases, red bars represent U-CLL and ID_high_ cases, green bars represent mutated IGHV (M-CLL) and ID_low_ cases, purple bars represent M-CLL and ID_high_ cases. *P* value refers to chi-square test.

According to the 98% cutoff, the whole cohort of 983 cases comprised 508 M- and 475 U-CLL with a distribution of IGHV genes comparable with literature data (Figure S11) [19]. Considering together ID and IGHV mutational status, we observed 422 ID_low_ U-CLL, 417 ID_low_ M-CLL, 53 ID_high_ U-CLL, and 92 ID_high_ M-CLL samples, with a significant overrepresentation of ID_high_ cases among M-CLL cases (*P* = *0.0028*, χ^2^ test; Fig. 4A), without a significant skewing in IGHV families and IGHV gene usage between ID_high_ and ID_low_ CLL (Fig. 4B, Figure S12, and Table S1). As reported in Table S1 and Table S3, we identified 111 out of 983 CLL (11.3%) belonging to the major IG stereotyped subsets [44]. Again, no evident skewing was observed between ID_high_ and ID_low_ in the context of CLL patients with stereotyped BCR (Table S3).

### Characterization of CLL with different ID levels: immunophenotype and cytogenetic lesions

Based on the analysis of immunophenotypic profiles in 189 CLL (112 ID_low_, 77 ID_high_ M-CLL), we showed no significant difference in term of MFI expression between ID_low_ and ID_high_ M-CLL, the majority of cases in both groups expressing a typical CLL immunophenotype (i.e. CD5+/CD23+/CD43+ with variable/dim expression of CD20/FMC7, as well as variable/dim expression of CD79b/SmIg/IgM; Figure S13) [27, 28].

Finally, no significant skewing was documented by comparing ID_high_ and ID_low_ cases (*n* = 840) in the context of the major cytogenetic lesions (i.e. del17p, del11q, del13q, and tris12; Table S1 and Table S4).

### Molecular mechanisms of ID in CLL

**Fig. 5:**
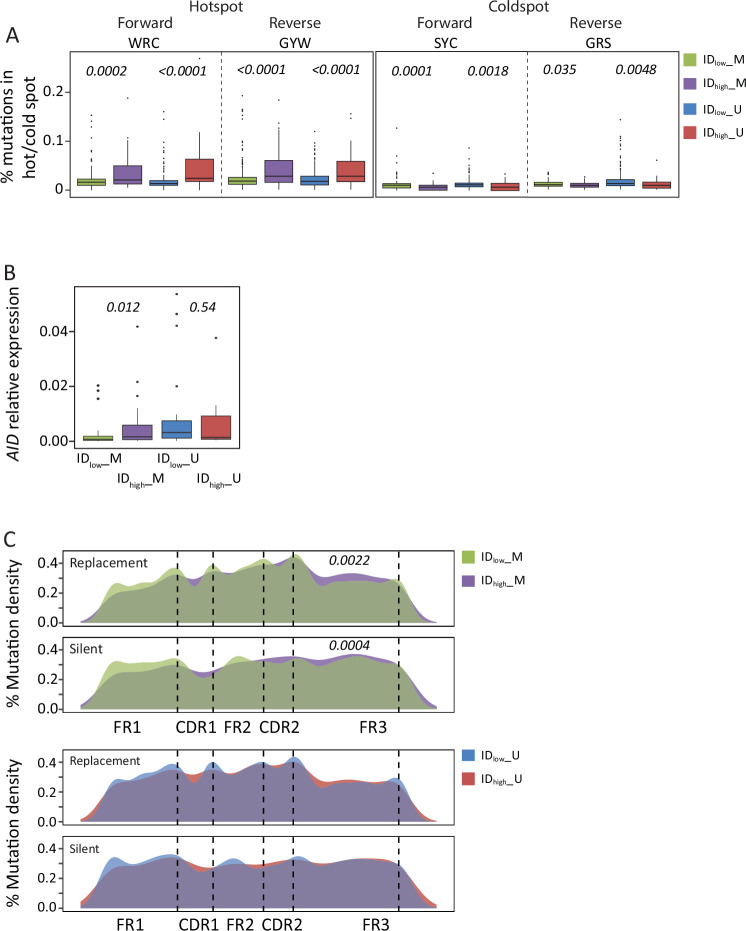
Evaluation of mutability rate. **A** Activation Induced Cytidine Deaminase (AID) activity. The boxplots on the right report the number of mutations compatible with AID mutational activity in both forward (WRC, W = A/T, R = A/G) and reverse strands (GYW, Y = C/T, W = A/T). The number of mutations occurring in AID coldspots for both the forward and reverse strand are reported on the left (SYC and GRS, S = G/C, Y = C/T, R = A/G). **B** The boxplots report the expression level of *AID* samples, 27 ID_high_ (19 M-, and 8 U-CLL) and 65 ID_low_ samples (40 M-, and 25 U-CLL). **C** The graphs report the density of replacement (R) and silent (S) mutations in dependence of the IGHV gene position. Green boxes represent M IGHV and ID_low_ cases, purple boxes represent M IGHV and ID_high_ cases. *P* values refer to student T-test.

Changes in mutability levels of know mutational hotspots/coldspots of AID and polymerase eta (Polη) [12, 22] were evaluated by processing 840 CLL samples amplified with IGHV Leader assay by means of the igphyml algorithm [24]. As shown in Fig. 5A, AID hotspots (WRC/GYW) significantly increased their mutability rates in the context of ID_high_ cases respect to ID_low_ cases, whilst AID coldspots (SYC/GRS), supposed not to be targeted by AID activity [12, 22], were significantly less mutated in ID_high_ samples respect to their ID_low_ counterpart.

By excluding all the shared mutation and circumscribing the analysis to the partially shared/unique mutations, i.e. the mutations allegedly acquired after the neoplastic transformation [11, 13], again a significant skewing of hotspot mutations was documented in ID_high_ M-CLL cases, indicative of an ongoing activity of the AID/Polη-dependent machinery (Figure S14).

To further validate the role of AID in determining ID in ID_high_ M-CLL, we evaluated *AICDA* expression levels in 90 samples, 27 ID_high_ (19 M-, and 8 U-CLL) and 65 ID_low_ samples (40 M-, and 25 U-CLL). As summarized in Fig. 5B, ID_high_ M-CLL expressed significantly higher transcript levels of *AID* compared to ID_low_ M-CLL samples (*P* = *0.012*), while no differences were observed in the U-CLL (*P* = 0.54). Despite a slight increase in median expression level of *AICDA* in ID_low_ U-CLL versus ID_high_ M-CLL (0.01 versus 0.0062 relative expressions) no significant difference was observed (*P* = 0.3040).

Finally, we evaluated the frequency of replacement and silent mutations along the IGHV sequence. In this regard, we showed a significant increase in the density of replacement mutations across the framework region 3 (FR3) in the context of ID_high_ M-CLL, while no differences were observed in the complementarity determining regions 1 and 2 (CDR1 and CDR2; Fig. 5C, and Figure S15).

Altogether, ID_high_ CLL, as identified according to our UMI-independent approach using the iSI cutoff, have features consistent with the physiologic AID-driven ID phenomenon occurring in normal B cell during the GC-specific B cell differentiation [45].

### Clonal evolution of ID over time

Sequential analysis of iSI scores, performed on 33 cases (21 classified as ID_high_ and 12 classified as ID_low_ CLL) revealed that all patients maintained the ID classification of the first available sample without changing the ID assignment (Figure S16). Comparing the first and last time point of each case no significant difference was observed in terms of iSI for both ID_high_ and ID_low_ (ID_high_: median first time point = 2.053, range 1.24–15.56, median last time point = 2.364, range 1.23–22.11; *P* = 0.340; ID_low_: median first time point = 1.027, range 1.00–1.12, median last time point = 1.044, range 1.00–1.16; *P* = 0.5181).

## Discussion

The present study took advantage from NGS IGHV sequencing data from a large CLL cohort generated during routine procedures and re-analyzed with a novel custom pipeline built ad-hoc to allow ID evaluation. To achieve this, we first developed an original bioinformatic pipeline to identify and correct systematic sequencing artifacts. In high-throughput parallel sequencing, the read quality lowers in a position-dependent and read-dependent fashion due to the decay of sequencing reagents [46, 47]. Randomic low-frequency errors may take place due to incorrect nucleotide incorporation of polymerases in both the PCR-amplification and the sequencing process. Moreover, it has been reported that data generated by the Miseq sequencer could be affected by systematic errors in dependence of the library-preparation protocol and the nucleotidic sequences flanking the specific base [46, 47]. Although not usually affecting mutation calculation in the context of the prevalent clone to define the M/U IGHV mutational status [48], systematic sequencing errors may have an impact on ID quantification. For this reason, we identified the specific nucleotidic sequences with the lowest quality score [46], and eventually corrected them by inserting a specific package in the pipeline of analysis.

Then, we moved to quantitatively estimate ID by taking advantage of the Hill number-based diversity profiles of diversity indices, a diversity metric borrowed from the ecology field and adapted to study the diversity repertoire of B-cell populations [36, 37, 49].

Here, instead of performing diversity measures on the heterogeneous B-cell population, we focused on diversity measures within the pathological clone, identified as the most expressed clone inside the sample bearing the same IGHV, IGHD, and IGHJ genes and identical/similar CDR3. In this context, subclones can be identified, defined as having the same IGHV, IGHD, IGHJ, and CDR3 sequence of the main clone but differing from the main clone by at least one nucleotide somewhere in the sequence; ID was defined by the presence of at least one subclone.

Among the different Hill number-derived indices, to quantify ID, we used the iSI, corresponding to a Hill number equal to 2, an index that allows the calculation of the intraclonal diversity by taking into consideration the proportional abundances of the different subclones within the immunoglobulin population in a UMI-independent and phylogenetic tree-independent approach [36, 37, 49].

Although the distribution of iSI in CLL was represented by a continuum, a significant 1.2 iSI cutoff was capable to discriminate CLL cases into two subsets with different TTFT intervals. By combining the conventional UM/M IGHV classification with a classification according to the 1.2 iSI cutoff, ID_high_ M-CLL displayed significantly longer TTFT than ID_low_ M-CLL patients, and ID emerged as an independent predictor of TTFT in M-CLL. Moreover, more complex phylogenetic tree-dependent parameters [17, 18, 20] were found in the context of ID_high_ CLL, and the majority of LPD characterized by frank ID features, i.e. HCL, DLBCL, and FL [38–42], displayed higher iSI respect to MCL usually displaying U-IGHV gene and lacking SHM features, given its alleged pre-GC origin [43, 50]. All together, these findings confirmed the validity of our approach which integrates information reported in the literature for ID identification with a new concept for ID quantification able to classify CLL based on the subclonal complexity without experimental-expensive or computing demanding workflows.

This is the largest CLL dataset of IGHV analyzed so far for ID and, according to our pipeline, about 15% of CLL patients had evidence of a high level of ID (the so-called ID_high_ CLL), in keeping with a previous report of ours [13]. Different percentages of ID_high_ cases, as identified in other papers [17, 51–54], were always related to very small and/or selected CLL cohorts. Overall, the highest percentage of ID_high_ cases (18.0%) was found in the context of M-CLL without a specific skewing in IGHV family/gene usage, and CLL subsets. Nevertheless, a non-negligible fraction of ID_high_ cases was also observed in U-CLL (11.1%), although in the absence of prognostic impact. The presence of a subset of ID_high_ U-CLL may be consistent with the post-GC derivation of (some) U-CLL, as historically reported [55, 56].

High mutability rates of AID/polη hotspots were detected in ID_high_ M-CLL, suggesting the involvement of a canonical AID-dependent SHM process in the in-vivo generation of ID in CLL, as preliminary observed by us in the pre-NGS era [13]. Consistently, higher *AICDA* levels in ID_high_ respect to ID_low_ M-CLL were demonstrated further underlining a direct involvement of this enzyme in the SHM process occurring in ID_high_ CLL. These findings are in keeping with Palacios et al. who reported a significant higher progression rate among M-CLL with null *AICDA* expression compared to M-CLL with higher *AICDA* expression, the latter allegedly resembling the AID-high/ID_high_ M-CLL from our cohort [57]. High *AICDA* levels were also detected in U-CLL, regardless of ID status, not significantly different from levels found in ID_high_ M-CLL. This observation, again consistent with previous reports [57–60], originally suggested a prominent role of AICD in ongoing class-switch recombination rather than SHM [58, 61]. The notion that high *AICDA* levels are also associated with high ongoing IGHV mutations in M-CLL, as shown here, suggested a more complete physiologic activity of *AICDA* in CLL, similar to that of normal germinal-center B cell reaction [40, 41, 59].

The reasons behind the better clinical outcome of AID-high/ID_high_ M-CLL remain to be established. A possible explanation could be related to a chronic ongoing antigen stimulation occurring in this CLL subset, in some instances associated with upregulation of AID and insertion of AID-driven ongoing mutations in IGHV genes, eventually leading to induction of an anergic CLL cell state and a better prognosis [62, 63]. Moreover, one can speculate that the ID process may contribute to increase a BCR-related generation of neoantigen expressed on CLL cells [38, 64], as suggested by a higher rate of replacement mutations in the FR3 region of ID_high_ M-CLL similarly seen in other LPD [64], with a subsequent more effective control of the CLL clone by reactive immune cells.

Altogether, evaluation of ID in CLL is feasible to be performed in the same context as the analysis of the IGHV gene mutational status and no other tests are required. In this scenario, an ad-hoc ID calculator will soon be available to calculate the iSI value and easily classify a given patient as ID_high_ or ID_low_.

In the present study, data of ID were mostly analyzed using the first available sample and always before the start of treatment. However, according to ID calculation in longitudinal samples, although generated on a small cohort again analyzed during the watch-and-wait CLL phase, it seems that a single determination is sufficient to classify CLL as ID_high_ or ID_low_.

Clinically, ID calculation may be useful to refine the CLL-IPI for the prediction of TTFT in CLL patients [65]. Furthermore, the clinical impact of ID evaluation in the context of new target therapies, including e.g. BCL-2 inhibitors for which it is known that the IGHV gene status still has a prognostic value [66, 67], will be our future goal.

In conclusion, here we were able to develop and validate a robust NGS protocol to quantitatively evaluate ID in CLL, demonstrating that a quantitative analysis of ID is feasible in a large-scale by a UMI-independent approach. In this context, ID has been virtually found, although at various degree, in all CLL patients. A high degree of ID was demonstrated to have a clinical impact by identifying a M-CLL subset with significantly better outcome.

## Supplementary information

Supplementary Materials

Supplementary Figures

Supplementary Tables

## Data Availability

Sequencing data are available upon reasonable request.

## References

[1] Eichhorst B, Ghia P, Niemann CU, Kater AP, Gregor M, Hallek M, et al. ESMO Clinical Practice Guideline interim update on new targeted therapies in the first line and at relapse of chronic lymphocytic leukaemia. Ann Oncol. 2024;35:762–8. 38969011 10.1016/j.annonc.2024.06.016

[2] Hallek M, Al-Sawaf O. Chronic lymphocytic leukemia: 2022 update on diagnostic and therapeutic procedures. Am J Hematol. 2021;96:1679–705. 34625994 10.1002/ajh.26367

[3] Shadman M. Diagnosis and treatment of chronic lymphocytic leukemia: a review. JAMA. 2023;329:918–32. 36943212 10.1001/jama.2023.1946

[4] Cohen JA, Bomben R, Pozzo F, Tissino E, Härzschel A, Hartmann TN, et al. An updated perspective on current prognostic and predictive biomarkers in chronic lymphocytic leukemia in the context of chemoimmunotherapy and novel targeted therapy. Cancers. 2020;12:894. 32272636 10.3390/cancers12040894 PMC7226446

[5] Lee J, Wang YL. Prognostic and predictive molecular biomarkers in chronic lymphocytic leukemia. J Mol Diagn. 2020;22:1114–25. 32615167 10.1016/j.jmoldx.2020.06.004

[6] International CLL-IPI working group. An international prognostic index for patients with chronic lymphocytic leukaemia (CLL-IPI): a meta-analysis of individual patient data. Lancet Oncol. 2016;17:779–90. 27185642 10.1016/S1470-2045(16)30029-8

[7] González-Gascón-Y-Marín I, Muñoz-Novas C, Rodríguez-Vicente A-E, Quijada-Álamo M, Hernández-Sánchez M, Pérez-Carretero C, et al. From biomarkers to models in the changing landscape of chronic lymphocytic leukemia: evolve or become extinct. Cancers. 2021;13:1–20. 10.3390/cancers13081782 PMC8068228 33917885

[8] Karr M, Roeker LA. History of targeted therapy development and progress in novel–novel combinations for chronic lymphocytic leukemia (CLL). Cancers. 2023;15. 10.3390/cancers15041018. 10.3390/cancers15041018 PMC9954076 36831364

[9] Patel K, Pagel JM. Current and future treatment strategies in chronic lymphocytic leukemia. J Hematol Oncol. 2021;14:69. 33902665 10.1186/s13045-021-01054-w PMC8074228

[10] Martin A, Chahwan R, Parsa JY, Scharff MD. Somatic Hypermutation: The Molecular Mechanisms Underlying the Production of Effective High-Affinity Antibodies. Elsevier 2015;20:363–88.

[11] Gurrieri C, McGuire P, Zan H, Yan XJ, Cerutti A, Albesiano E, et al. Chronic lymphocytic leukemia B cells can undergo somatic hypermutation and intraclonal immunoglobulin VHDJH gene diversification. J Exp Med. 2002;196:629–39. 12208878 10.1084/jem.20011693 PMC2194006

[12] Pilzecker B, Jacobs H. Mutating for good: DNA damage responses during somatic hypermutation. Front Immunol. 2019;10:438. 30915081 10.3389/fimmu.2019.00438 PMC6423074

[13] Degan M, Bomben R, Bo MD, Zucchetto A, Nanni P, Rupolo M, et al. Analysis of IgV gene mutations in B cell chronic lymphocytic leukaemia according to antigen-driven selection identifies subgroups with different prognosis and usage of the canonical somatic hypermutation machinery. Br J Haematol. 2004;126:29–42. 15198729 10.1111/j.1365-2141.2004.04985.x

[14] Scheijen B, Meijers RWJ, Rijntjes J, van der Klift MY, Möbs M, Steinhilber J, et al. Next-generation sequencing of immunoglobulin gene rearrangements for clonality assessment: a technical feasibility study by EuroClonality-NGS. Leukemia. 2019;33:2227–40. 31197258 10.1038/s41375-019-0508-7 PMC6756030

[15] Khan TA, Friedensohn S, Gorter de Vries AR, Straszewski J, Ruscheweyh H-J, Reddy ST. Accurate and predictive antibody repertoire profiling by molecular amplification fingerprinting. Sci Adv. 2016;2:e1501371. 26998518 10.1126/sciadv.1501371 PMC4795664

[16] Turchaninova MA, Davydov A, Britanova OV, Shugay M, Bikos V, Egorov ES, et al. High-quality full-length immunoglobulin profiling with unique molecular barcoding. Nat Protoc. 2016;11:1599–616. 27490633 10.1038/nprot.2016.093

[17] Bagnara D, Tang C, Brown JR, Kasar S, Fernandes S, Colombo M, et al. Post-transformation IGHV-IGHD-IGHJ mutations in chronic lymphocytic leukemia B cells: implications for mutational mechanisms and impact on clinical course. Front Oncol. 2021;11:1–14. 10.3389/fonc.2021.640731 PMC8186829 34113563

[18] Zaragoza-Infante L, Junet V, Pechlivanis N, Fragkouli S-C, Amprachamian S, Koletsa T, et al. IgIDivA: immunoglobulin intraclonal diversification analysis. Brief Bioinform. 2022;23:1–11. 10.1093/bib/bbac349 PMC9487589 36044248

[19] Agathangelidis A, Chatzidimitriou A, Gemenetzi K, Giudicelli V, Karypidou M, Plevova K, et al. Higher-order connections between stereotyped subsets: implications for improved patient classification in CLL. Blood. 2021;137:1365–76. 32992344 10.1182/blood.2020007039 PMC7976441

[20] Jeusset L, Abdollahi N, Verny T, Armand M, De Septenville AL, Davi F, et al. ViCloD, an interactive web tool for visualizing B cell repertoires and analyzing intraclonal diversities: application to human B-cell tumors. NAR Genom Bioinforma. 2023;5:lqad064. 10.1093/nargab/lqad064 PMC10304752 37388820

[21] Bystry V, Agathangelidis A, Bikos V, Sutton LA, Baliakas P, Hadzidimitriou A, et al. ARResT/AssignSubsets: a novel application for robust subclassification of chronic lymphocytic leukemia based on B cell receptor IG stereotypy. Bioinformatics. 2015;31:3844–6. 26249808 10.1093/bioinformatics/btv456

[22] Pettersen HS, Galashevskaya A, Doseth B, Sousa MML, Sarno A, Visnes T, et al. AID expression in B-cell lymphomas causes accumulation of genomic uracil and a distinct AID mutational signature. DNA Repair (Amst). 2015;25:60–71. 25486549 10.1016/j.dnarep.2014.11.006

[23] Maura F, Degasperi A, Nadeu F, Leongamornlert D, Davies H, Moore L, et al. A practical guide for mutational signature analysis in hematological malignancies. Nat Commun. 2019;10:2969. 31278357 10.1038/s41467-019-11037-8 PMC6611883

[24] Hoehn KB, Vander Heiden JA, Zhou JQ, Lunter G, Pybus OG, Kleinstein SH. Repertoire-wide phylogenetic models of B cell molecular evolution reveal evolutionary signatures of aging and vaccination. Proc Natl Acad Sci USA. 2019;116:22664–72. 31636219 10.1073/pnas.1906020116 PMC6842591

[25] Catovsky D, Richards S, Matutes E, Oscier D, Dyer M, Bezares RF, et al. Assessment of fludarabine plus cyclophosphamide for patients with chronic lymphocytic leukaemia (the LRF CLL4 Trial): a randomised controlled trial. Lancet. 2007;370:230–9. 17658394 10.1016/S0140-6736(07)61125-8

[26] Oscier D, Wade R, Davis Z, Morilla A, Best G, Richards S, et al. Prognostic factors identified three risk groups in the LRF CLL4 trial, independent of treatment allocation. Suppl Haematol. 2010;95:1705–12. 10.3324/haematol.2010.025338 PMC2948096 20511662

[27] Tissino E, Pozzo F, Benedetti D, Caldana C, Bittolo T, Rossi FM, et al. CD49d promotes disease progression in chronic lymphocytic leukemia: new insights from CD49d bimodal expression. Blood. 2020;135:1244–54. 32006000 10.1182/blood.2019003179 PMC7228464

[28] Tissino E, Benedetti D, Herman SEM, Ten Hacken E, Ahn IE, Chaffee KG, et al. Functional and clinical relevance of VLA-4 (CD49d/CD29) in ibrutinib-treated chronic lymphocytic leukemia. J Exp Med. 2018;215:681–97. 29301866 10.1084/jem.20171288 PMC5789417

[29] Bland JM, Altman DG. Measuring agreement in method comparison studies. Stat Methods Med Res. 1999;8:135–60. 10501650 10.1177/096228029900800204

[30] Hothorn T, Lausen B. On the exact distribution of maximally selected rank statistics. Comput Stat Data Anal. 2003;43:121–37.

[31] Ciampi A, Lawless JF, McKinney SM, Singhal K. Regression and recursive partition strategies in the analysis of medical survival data. J Clin Epidemiol. 1988;41:737–48. 3418363 10.1016/0895-4356(88)90160-6

[32] Lefranc M-P, Giudicelli V, Duroux P, Jabado-Michaloud J, Folch G, Aouinti S, et al. IMGT®, the international ImMunoGeneTics information system® 25 years on. Nucleic Acids Res. 2015;43:D413–22. 25378316 10.1093/nar/gku1056 PMC4383898

[33] Rognes T, Flouri T, Nichols B, Quince C, Mahé F. VSEARCH: a versatile open source tool for metagenomics. PeerJ. 2016;4:e2584. 27781170 10.7717/peerj.2584 PMC5075697

[34] Martin M. Cutadapt removes adapter sequences from high-throughput sequencing reads. EMBnet J. 2011;17:10.

[35] Ye J, Ma N, Madden TL, Ostell JM. IgBLAST: an immunoglobulin variable domain sequence analysis tool. Nucleic Acids Res. 2013;41. 10.1093/NAR/GKT382. 10.1093/nar/gkt382 PMC3692102 23671333

[36] Chao A, Gotelli NJ, Hsieh TC, Sander EL, Ma KH, Colwell RK, et al. Rarefaction and extrapolation with Hill numbers: a framework for sampling and estimation in species diversity studies. Ecol Monogr. 2014;84:45–67.

[37] Stern JNH, Yaari G, Vander Heiden JA, Church G, Donahue WF, Hintzen RQ, et al. B cells populating the multiple sclerosis brain mature in the draining cervical lymph nodes. Sci Transl Med. 2014;6:248ra107. 10.1126/scitranslmed.3008879 PMC4388137 25100741

[38] Xu-Monette ZY, Li J, Xia Y, Crossley B, Bremel RD, Miao Y, et al. Immunoglobulin somatic hypermutation has clinical impact in DLBCL and potential implications for immune checkpoint blockade and neoantigen-based immunotherapies. J Immunother Cancer. 2019;7:272. 31640780 10.1186/s40425-019-0730-x PMC6806565

[39] Forconi F, Sahota SS, Raspadori D, Ippoliti M, Babbage G, Lauria F, et al. Hairy cell leukemia: at the crossroad of somatic mutation and isotype switch. Blood. 2004;104:3312–7. 15284115 10.1182/blood-2004-03-0950

[40] Arons E, Roth L, Sapolsky J, Suntum T, Stetler-Stevenson M, Kreitman RJ. Evidence of canonical somatic hypermutation in hairy cell leukemia. Blood. 2011;117:4844–51. 21368287 10.1182/blood-2010-11-316737 PMC3100693

[41] Spence JM, Abumoussa A, Spence JP, Burack WR. Intraclonal diversity in follicular lymphoma analyzed by quantitative ultradeep sequencing of noncoding regions. J Immunol. 2014;193:4888–94. 25311808 10.4049/jimmunol.1401699 PMC4225181

[42] Leich E, Maier C, Bomben R, Vit F, Bosi A, Horn H, et al. Follicular lymphoma subgroups with and without t(14;18) differ in their N-glycosylation pattern and IGHV usage. Blood Adv. 2021;5:4890–900. 34614504 10.1182/bloodadvances.2021005081 PMC9153045

[43] Stevenson FK, Sahota SS, Ottensmeier CH, Zhu D, Forconi F, Hamblin TJ. The occurrence and significance of V gene mutations in B cell-derived human malignancy. Adv Cancer Res. 2001;83:81–116. 11665722 10.1016/s0065-230x(01)83004-9

[44] Agathangelidis A, Chatzidimitriou A, Chatzikonstantinou T, Tresoldi C, Davis Z, Giudicelli V, et al. Immunoglobulin gene sequence analysis in chronic lymphocytic leukemia: the 2022 update of the recommendations by ERIC, the European Research Initiative on CLL. Leukemia. 2022;36:1961–8. 35614318 10.1038/s41375-022-01604-2 PMC9343247

[45] Wu X, Feng J, Komori A, Kim EC, Zan H, Casali P. Immunoglobulin somatic hypermutation: double-strand DNA breaks, AID and error-prone DNA repair. J Clin Immunol. 2003;23:235–46. 12959216 10.1023/a:1024571714867 PMC4624321

[46] Schirmer M, Ijaz UZ, D’Amore R, Hall N, Sloan WT, Quince C. Insight into biases and sequencing errors for amplicon sequencing with the Illumina MiSeq platform. Nucleic Acids Res. 2015;43:e37. 25586220 10.1093/nar/gku1341 PMC4381044

[47] Kozich JJ, Westcott SL, Baxter NT, Highlander SK, Schloss PD. Development of a dual-index sequencing strategy and curation pipeline for analyzing amplicon sequence data on the MiSeq Illumina sequencing platform. Appl Environ Microbiol. 2013;79:5112–20. 23793624 10.1128/AEM.01043-13 PMC3753973

[48] Davi F, Langerak AW, de Septenville AL, Kolijn PM, Hengeveld PJ, Chatzidimitriou A, et al. Immunoglobulin gene analysis in chronic lymphocytic leukemia in the era of next generation sequencing. Leukemia. 2020;34:2545–51. 32561841 10.1038/s41375-020-0923-9 PMC7515836

[49] Gupta NT, Vander Heiden JA, Uduman M, Gadala-Maria D, Yaari G, Kleinstein SH. Change-O: a toolkit for analyzing large-scale B cell immunoglobulin repertoire sequencing data. Bioinformatics. 2015;31:3356–8. 26069265 10.1093/bioinformatics/btv359 PMC4793929

[50] Hardin CC, Armitage JO, Longo DL. Mantle-cell lymphoma. N Engl J Med. 2022;386:2495–506. 35767440 10.1056/NEJMra2202672

[51] Volkheimer AD, Weinberg JB, Beasley BE, Whitesides JF, Gockerman JP, Moore JO, et al. Progressive immunoglobulin gene mutations in chronic lymphocytic leukemia: evidence for antigen-driven intraclonal diversification. Blood. 2007;109:1559–67. 17082314 10.1182/blood-2006-05-020644 PMC1794047

[52] Bagnara D, Callea V, Stelitano C, Morabito F, Fabris S, Neri A, et al. IgV gene intraclonal diversification and clonal evolution in B-cell chronic lymphocytic leukaemia. Br J Haematol. 2006;133:50–8. 16512828 10.1111/j.1365-2141.2005.05974.x

[53] Kostareli E, Sutton LA, Hadzidimitriou A, Darzentas N, Kouvatsi A, Tsaftaris A, et al. Intraclonal diversification of immunoglobulin light chains in a subset of chronic lymphocytic leukemia alludes to antigen-driven clonal evolution. Leukemia. 2010;24:1317–24. 20463750 10.1038/leu.2010.90

[54] Sutton LA, Kostareli E, Hadzidimitriou A, Darzentas N, Tsaftaris A, Anagnostopoulos A, et al. Extensive intraclonal diversification in a subgroup of chronic lymphocytic leukemia patients with stereotyped IGHV4-34 receptors: Implications for ongoing interactions with antigen. Blood. 2009;114:4460–8. 19713457 10.1182/blood-2009-05-221309

[55] Damle RN, Ghiotto F, Valetto A, Albesiano E, Fais F, Yan XJ, et al. B-cell chronic lymphocytic leukemia cells express a surface membrane phenotype of activated, antigen-experienced B lymphocytes. Blood. 2002;99:4087–93. 12010811 10.1182/blood.v99.11.4087

[56] Klein U, Tu Y, Stolovitzky GA, Mattioli M, Cattoretti G, Husson H, et al. Gene expression profiling of B cell chronic lymphocytic leukemia reveals a homogeneous phenotype related to memory B cells. J Exp Med. 2001;194:1625–38. 11733577 10.1084/jem.194.11.1625 PMC2193527

[57] Palacios F, Moreno P, Morande P, Abreu C, Correa A, Porro V, et al. High expression of AID and active class switch recombination might account for a more aggressive disease in unmutated CLL patients: link with an activated microenvironment in CLL disease. Blood. 2010;115:4488–96. 20233972 10.1182/blood-2009-12-257758

[58] Oppezzo P, Vuillier F, Vasconcelos Y, Dumas G, Magnac C, Payelle-Brogard B, et al. Chronic lymphocytic leukemia B cells expressing AID display dissociation between class switch recombination and somatic hypermutation. Blood. 2003;101:4029–32. 12521993 10.1182/blood-2002-10-3175

[59] Albesiano E, Messmer BT, Damle RN, Allen SL, Rai KR, Chiorazzi N. Activation-induced cytidine deaminase in chronic lymphocytic leukemia B cells: expression as multiple forms in a dynamic, variably sized fraction of the clone. Blood. 2003;102:3333–9. 12855567 10.1182/blood-2003-05-1585

[60] Heintel D, Kroemer E, Kienle D, Schwarzinger I, Gleiß A, Schwarzmeier J, et al. High expression of activation-induced cytidine deaminase (AID) mRNA is associated with unmutated IGVH gene status and unfavourable cytogenetic aberrations in patients with chronic lymphocytic leukaemia. Leukemia. 2004;18:756–62. 14961036 10.1038/sj.leu.2403294

[61] Cerutti A, Zan H, Kim EC, Shah S, Schattner EJ, Schaffer A, et al. Ongoing in vivo immunoglobulin class switch DNA recombination in chronic lymphocytic leukemia B cells. J Immunol. 2002;169:6594–603. 12444172 10.4049/jimmunol.169.11.6594 PMC4625981

[62] Packham G, Krysov S, Allen A, Savelyeva N, Steele AJ, Forconi F, et al. The outcome of B-cell receptor signaling in chronic lymphocytic leukemia: proliferation or anergy. Haematologica. 2014;99:1138–48. 24986876 10.3324/haematol.2013.098384 PMC4077074

[63] Tan C, Noviski M, Huizar J, Zikherman J. Self-reactivity on a spectrum: a sliding scale of peripheral B cell tolerance. Immunol Rev. 2019;292:37–60. 31631352 10.1111/imr.12818 PMC6935424

[64] Khodadoust MS, Olsson N, Chen B, Sworder B, Shree T, Liu CL, et al. B-cell lymphomas present immunoglobulin neoantigens. Blood. 2019;133:878–81. 30545830 10.1182/blood-2018-06-845156 PMC6384186

[65] Pflug N, Bahlo J, Shanafelt TD, Eichhorst BF, Bergmann MA, Elter T, et al. Development of a comprehensive prognostic index for patients with chronic lymphocytic leukemia. Blood. 2014;124:49–62. 24797299 10.1182/blood-2014-02-556399 PMC4260976

[66] Allan JN, Flinn IW, Siddiqi T, Ghia P, Tam CS, Kipps TJ, et al. Outcomes in patients with high-risk features after fixed-duration ibrutinib plus venetoclax: phase II CAPTIVATE study in first-line chronic lymphocytic Leukemia. Clin Cancer Res. 2023;29:2593–601. 37282671 10.1158/1078-0432.CCR-22-2779 PMC10345960

[67] Al-Sawaf O, Robrecht S, Zhang C, Olivieri S, Chang YM, Fink AM, et al. Venetoclax-obinutuzumab for previously untreated chronic lymphocytic leukemia: 6-year results of the randomized phase 3 CLL14 study. Blood. 2024;144:1924–35. 39082668 10.1182/blood.2024024631 PMC11551846

