# Supplementary material for: Measurement of Immunoglobulin Intraclonal diversification refines the clinical impact of IGHV mutational status in chronic lymphocytic leukemia
Source: Leukemia. 2025 Jun 18;39(8):1905–14. doi: 10.1038/s41375-025-02650-2 (PMC12310548; doi:10.1038/s41375-025-02650-2)
Supplement: Supplementary file 1 — Supplementary Materials [file 41375_2025_2650_MOESM1_ESM.docx]

**Supplemental Information**

**Measurement of Immunoglobulin Intraclonal diversification refines the clinical impact of IGHV mutational status in chronic lymphocytic leukemia**

Filippo Vit^1^, Tamara Bittolo^1^, Antonella Zucchetto^1^, Robel Papotti^1^, Erika Tissino^1^, Federico Pozzo^1^, Annalisa Gaglio^1^, Andrea Stacchetti^1^, Eva Zaina^1^, Ilaria Cattarossi^1^, Paola Varaschin^1^, Paola Nanni^1^, Michele Berton^1^, Alessandra Braida^1^, Francesca Maria Rossi^1^, Massimo Degan^1^, Jerry Polesel^2^, Roberta Laureana^1,3^, Annalisa Chiarenza^4^, Jacopo Olivieri^5^, Annalisa Biagi^6^, Giovanni D’Arena^7^, Marco Rossi^8^, Luca Laurenti^9^, Agostino Tafuri^10^, Pietro Bulian^1^, Alberto Zamò^11^, Ellen Leich^11^, Andreas Rosenwald^11^, Evgeny Arons^12^, Robert Kreitman^12^, Massimo Gentile^13,14^, Massimiliano Postorino^3^, Francesco Zaja^15^, Francesco Di Raimondo^4^, Maria Ilaria Del Principe^3^, Valter Gattei^1*^, and Riccardo Bomben^1*^.

^1^Clinical and Experimental Onco-Haematology Unit, Centro di Riferimento Oncologico di Aviano (CRO), IRCCS, Aviano (PN), Italy;

^2^Unit of Cancer Epidemiology, Centro di Riferimento Oncologico di Aviano (CRO) IRCCS, Aviano, Italy;

^3^Division of Hematology, University of Tor Vergata, Rome, Italy;

^4^Division of Haematology, Ferrarotto Hospital, Catania, Italy;

^5^Clinica Ematologica, Centro Trapianti e Terapie Cellulari "Carlo Melzi" DISM, Azienda Ospedaliera Universitaria S. Maria Misericordia, Udine, Italy;

^6^Hematology and Transplant Unit, Ospedale S.M.Goretti, AUSL Latina, Italy;

^7^Haematology Unit, Presidio Ospedaliero S. Luca, ASL Salerno, Italy;

^8^Department of Hematology-Oncology, Dulbecco University Hospital, 88100 Catanzaro, Italy;

^9^Fondazione Universitaria Policlinico A Gemelli di Roma, Roma, Italy;

^10^Department of Clinical and Molecular Medicine and Hematology, Sant'Andrea - University Hospital - Sapienza, University of Rome, Rome, Italy;

^11^Institute of Pathology, University of Würzburg, Würzburg, Germany;

^12^Center for Cancer Research, National Cancer Institute, Bethesda, MD, USA;

^13^Hematology Unit, Azienda Ospedaliera Annunziata, Cosenza, Italy;

^14^Department of Pharmacy, Health and Nutritional Science, University of Calabria, Rende, Italy;

^15^Department of Medical, Surgical and Health Sciences, University of Trieste, Italy;

*Equally contributed as senior authors.

**Correspondence:** Filippo Vit or Valter Gattei or Riccardo Bomben, Clinical and Experimental Onco-Hematology Unit, Centro di Riferimento Oncologico, I.R.C.C.S., Via Franco Gallini 2, Aviano (PN), Italy; email: [filippo.vit@cro.it](mailto:filippo.vit@cro.it); [vgattei@cro.it](mailto:vgattei@cro.it); [rbomben@cro.it](mailto:rbomben@cro.it).

- - **Supplemental Figures:**
- **Figure S1. Library generation protocol for Immunoglobulin amplification with Unique Molecular Identifiers (UMI);**
- **Figure S2. Flow-chart representation of the CLL samples analyzed in this study;**
- **Figure S3. Bioinformatic pipeline for Lymphotrack RepSeq data;**
- **Figure S4. Read quality profile of Lymphotrack RepSeq data;**
- **Figure S5. Identification of systematic error based on median Phred quality;**
- **Figure S6. Schematic representation of the systematic sequencing errors (SSE) correction pipeline;**
- **Figure S7. Evaluation of the error-correction pipeline in IGHV-Leader and FR1 and comparison between Unique Molecular Identifier (UMI) and IGHV Leader protocol;**
- **Figure S8. Schematic representation of the bioinformatic pipeline for the analysis of RepSeq data tagged with Unique Molecular Identifiers (UMI);**
- **Figure S9. IGHV mutational status in the total cohort;**
- **Figure S10. Pairwise comparisons between phylogenetic tree parameters;**
- **Figure S11. Distribution of IGHV families and genes in the CLL cohort;**
- **Figure S12. Top 10 IGHV genes with high intraclonal diversification (ID) among U and M-CLL;**
- **Figure S13. Immunophenotypic analysis of cases with high intraclonal diversification (ID) in M-CLL;**
- **Figure S14. Variation in the number of mutations compatible with Activation Induced Cytidine Deaminase (AID) and polymerase eta;**
- **Figure S15. Variation in percentage of replacement and silent mutations in the FR and CDR region;**
- **Figure S16. Clonal evolution of ID in longitudinal ID_high_ samples.**

**Supplemental Tables:**

- **Table S1. IGHV-D-J usage and intraclonal diversification;**
- **Table S2. Primers for IGHV UMI analysis.**

**Supplementary Material**

**Immunophenotypic analyses**

Expression of CLL markers was evaluated by flow cytometry on fresh peripheral blood (PB) CLL samples as part of the routine diagnostic procedures for CLL assessment using a FACSCantoII (BD Biosciences, La Jolla, CA, USA) flow cytometer after calibration of the instrument with CS&T beads (BD Biosciences), using the FACSDiva software (BD Biosciences). The analysis was limited to samples collected between August 2015 and August 2018, for which the same panel of antibodies was used, allowing data comparability. The monoclonal antibodies (mAbs) used were CD19 PerCP-Cy5.5, CD5 FITC, CD43 APC-H7, CD20 PE-Cy7, CD23 APC, CD79b APC (all from BD Biosciences) and surface membrane immunoglobulin (SmIg) Kappa light chain PE and Lambda FITC (Dako). For all molecules, expression data was collected as mean fluorescence intensity (MFI) in the context of the CD5+CD19+ population with the exception of SmIg expression which was analysed in the context of the CD19+ population.

**IGHV sequencing**

DNA and RNA of CLL cases were obtained from fresh buffy coats following commercially available extraction protocols (NucleoSpin Blood kit, Macherey-Nagel for DNA and Trizol, ThermoFischer for RNA) according to manufacturer instructions. Total RNA (500 ng) was retro-transcribed with the Improm-II Reverse Transcription System (Promega) using polyT-specific primers following manufacturer instructions. Sequencing analysis of IGHV was performed on either genomic DNA or complementary DNA (cDNA) using consensus primers for the IGHV leader or the IGHV FR1 regions in conjunction with JH primers, according to LymphoTrack IGHV Leader or IGHV FR1 assays (Invivoscribe), as previously reported.^1^ Sequences were analyzed using the IMGT databases and the IMGT/V-QUEST tool (<http://imgt.org/>, version 3.2.17, Université Montpellier 2, CNRS, LIGM, Montpellier, France).^2^ Sequences with ≥98% identity to the germline were considered U-IGHV; sequences with <98% identity to the germline were considered M-IGHV.^3–8^ As summarized in Figure 1 and Figure S2, IGHV libraries were generated in 923 samples using IGHV Leader-specific primers starting from cDNA, while in 168 samples IGHV FR1-specific primers were employed to amplify DNA. The LymphoTrack IGHV FR1 assay was also used for libraries generation from DNA of DLBCL, FL, HCL and MCL. After purification (PureLink Quick PCR Purification kit, ThermoFischer) and dilution, PCR products were sequenced. In all cases, sequencing was performed on a MiSeq (Illumina).

**Immunoglobulin sequencing library analysis**

To perform the analysis of the repertoire sequencing (RepSeq) data of the heavy chain variable gene (IGHV) generated with LymphoTrack^1^ we designed a specific custom pipeline and applied it to 1,091 CLL samples (Figure S2 and S3).

In detail, the pipeline consisted of two parts (Figure S3): i) the first part adopted canonical steps generally employed in all RepSeq analysis;^9–12^ ii) the second part was tailored to handle systematic errors, correct them, and analyze data to provide a measurement for the intraclonal diversification, i.e. the diversity inside the pathological clone through calculation of the inverse Simpson Index (iSI).^12,13^ The pathological clone was identified as the most expressed clone inside the sample: each sequence with the same variable (IGHV), diversity (IGHD) and junction (IGHJ) segments and bearing almost identical complementary determining region 3 (CDR3) were considered as belonging to the same clone. A subclone was defined as having the same IGHV, IGHD, IGHJ and CDR3 sequence of the main clone with at least 1 nucleotide difference (intraclonal diversification, ID).

**General pipeline for RepSeq analysis**

The pipeline is summarized in Figure S3. Briefly, fastq were demultiplexed with bcl2fastq (v. 2.6.1). Paired fastq reads were merged and hard filtered with vsearch (v.2.14.2).^14^ Merging and filtering steps were performed with loose parameters (--fastq_mergepairs -fastq_minmergelen 5 -fastq_maxdiffs 20; --fastq_filter -fastq_minlen 100 -fastq_maxee 3.0) to keep most of the data for further analysis. Primers were removed with cutadapt (v. 2.3) and reads with no primers found were removed.^15^ Residual reads were collapsed with a custom python script and those with a read count equal to 1 were removed. Remaining reads were aligned with IgBlast (v.1.8.0) against the IMGT reference database (updated August 2022; http://imgt.org/).^2,11^ The remaining sequences were filtered to keep functional Ig sequences only with a custom python script. Clonotype assignment and germline identification were performed with the ChangeO package (v.0.4.6).^10^ We exploited the DefineClones.py package of ChangeO which assigns clonotypes based on IGHV, IGHJ and similar IGHV complementarity region 3 (CDR3) usages. In particular, sequences with same IGHV, IGHJ, and same-length CDR3 with a maximum nucleotidic Hamming distance equal to 0.2 were assigned to the same clonotype/clone. Accordingly, each clonotype/clone consists of all descendants (subclones) of a single, fully rearranged common ancestor, as the result of the somatic hypermutation (SHM) process introducing mutations at any position, thus yielding multiple VDJ sequence transcripts (subclones), all deriving from a specific clonotype.

This part of the analysis produces three files: 1) *MajClone.tab reporting clonotypes with their relative frequencies; 2) *germ-pass.tab, the ChangeO-generated.tab delimited file with all the information regarding Ig analyzed; 3) grouped.fastq which is a fastq file containing all the reads survived from merging, filtering and primer-removal steps.

All the subsequent analyses were performed only on the sequences belonging to the pathological clone identified as reported above.

**Identification of systematic error, systematic error correction pipeline and ID measurement**

The second part of the analysis reported below (Systematic sequencing errors correction pipeline; Figure S3) exploits information contained in all the three files generated as reported above to correct systematic sequencing errors and eventually calculated the iSI as a measure of ID.

***i) Identification of systematic sequencing errors***

We calculated the median Phred quality of single nucleotides in dependence of the read position and tri-nucleotidic motif before. In keeping with Schirmer et. al.^16^ we observed a global decrease in Read2 (R2) quality respect to Read1 (R1) in the context of experiments carried out using the Illumina MiSeq sequencer; in particular, we found huge drops associated with specific trinucleotidics sequences in dependence of particular read position especially in R2. As shown in Figure S4AC, the heatmaps of the median Phred quality observed in dependence of i) the number of cycles, ii) nucleotide observed, and iii) preceding trinucleotidic motifs is different by comparing MiSeq Read1 and MiSeq Read2, the overall quality being reduced in Read2. Lower mean quality score was observed more frequently in the context of FR3 region of IGHV4 and IGHV5 respect to the others IGHV families, due to the particular trimers composition of these 2 families (Figure S4C).

To discriminate between true mutations and systematic sequencing errors, we selected 62 samples previously amplified with the LymphoTrack IGHV leader assay and we re-processed them with the LymphoTrack IGHV FR2 assay that allows generating completely superimposable MiSeq paired reads (R1, and R2). We exploited the full reads superimposition of the FR2 assay to evaluate whether mutations observed in the same region of samples processed by using the LymphoTrack IGHV leader assay were completely recapitulated. Mutations (respect to the assigned germline) were identified on re-aligned immunoglobulin fastq sequences (see below) and mutational frequency was calculated as the ratio between the numbers of the alternate alleles respect to the total observations. Based on a frequency range of 0.1%-100%, we identified 3,021, and 1,672 mutations respectively using the IGHV leader and the IGHV FR2 assay with 1,672 mutations commonly identified by both protocols (Figure S5A). Interestingly, 338 out of 1,349 mutations only identified by the IGHV leader assay had frequencies higher than 1% (range 1%-61%, median 3.13%) that could massively affect the iSI calculation and ID measurement (see below). We then calculated the Phred quality score for each mutation by means of the vsearch algorithm. In the context of the median Phred for the 3,021 mutations, a significantly lower Phred score was observed for the 1,349 mutations identified only with LymphoTrack leader assay respect to the 1,672 common mutations (*P<0.0001*; Figure S5B). These findings are in keeping with Kozich et al. who identified systematic errors as point mutations with Phred quality <21.^17^ Based on these data, we integrated this information in our custom pipeline to correct sequencing errors.

***ii) Systematic sequencing errors (SSE) correction pipeline***

The SSE pipeline, summarized in Figure S3, is an original pipeline (Italian Patent n.102022000027138_15422) made of 5 different packages: realign-reads.py, Ig-Mutations_parsing_from-unique.py, Parse_Ig_QUAL.py, reCollapse.py and iSI_calculation.py. Realign-reads.py takes original fastq data, selects reads belonging to the pathological clone and realigns them against the IMGT-gapped germline sequences and produces a .fasta file whose sequences are aligned according the IMGT numeration. Ig-Mutations_parsing_from-unique.py package takes as input the aligned .fasta file and generates a pileup file consisting in a NxM table (with N=[A, C, G, T], all the possible nucleotides, M=[1...n], all the Ig positions (Figure S3). The Parse_Ig_QUAL.py is responsible for the SSE identification and correction. Briefly, to identify SSE the pileup generated in the previous step is parsed and all the positions with a cumulative frequency >=0.1 are considered. If the median quality observed in dependence of the Ig-position is lower than 21 it is considered as a SSE and corrected. The erroneous nucleotide is substituted with the second most expressed nucleotide in that position with the highest median quality (figure S6). ReCollapse.py re-collapses the newly corrected sequences and iSI_calculation.py calculates the iSI on corrected sequences.

***iii) Inverse Simpson index (iSI) calculation***

See main text.

**IGHV Leader and FR1 assays comparison**

As summarized in Figure S2, IGHV libraries were generated in 923 samples using IGHV Leader-specific primers starting from cDNA, while in 168 samples IGHV FR1-specific primers were employed to amplify DNA. The LymphoTrack IGHV FR1 assay was also used for libraries generation from DNA of DLBCL, FL, HCL, and MCL.

To demonstrate that iSI calculation would provide superimposable results when analyzing libraries generated from IGHV Leader assays and/or IGHV FR1 assays, we amplified 2 ul of cDNA and 100 ng of DNA of 91 CLL samples, all with a single and identifiable pathological clone and different levels of ID, both with LymphoTrack IGHV Leader and IGHV FR1 assays, respectively. Libraries were sequenced on different flow cells to avoid possible errors in index assignment. IGHV Leader/FR1 data were processed with our custom pipeline for error correction and sequence frequencies were compared. Identification of the pathological clone was performed as reported above, and accordingly all the immunoglobulin sequences different in terms of nucleotidic sequence were identified as subclones. We then generated a python script which clusters sequences between IGHV Leader and FR1 data with a 100% identity through the DBSCAN algorythm of sklearn python package.^18,19^ Both IGHV Leader and FR1 corrected sequences were given as arguments for the script.

**UMI-tagged Ig library generation**

To compare the results generated by our custom pipeline against the gold standard for the RepSeq analysis, we adapted an Ig library preparation protocol exploiting Unique Molecular Identifier (UMI)-tagged primers (Figure S1).^20^ Firstly, we amplified 500 ng of RNA with a JH-specific UMI-tagged RT-primer to specifically retrotranscribe only Ig sequences. Since having clonotype information from LymphoTrack generated libraries, we used single IGHV-specific primers (Table S2) to avoid amplification biases in multiplex PCR reactions. A single-cycle PCR was adopted to insert the IGHV-specific UMI-tagged primer with the specific programs (1 cycle of 98°C for 30 s; 55°C for 2 min; 72°C for 15 min) using a Verity Thermal Cycler (ThermoFischer). Amplicon quantification was performed with an in-house qPCR assay with custom primers for Illumina partial adapter (fwd: GTTCTACAGTCCGACGATCG, rev: TTGGCACCCGAGAATTCCAC). Then, 30.000 Ig-molecules, to avoid excessive Ig singletons that could affect the analysis, was used for the second round PCR and indexed with custom primers containing P5 and P7 Illumina sequences adapters (Table S2), with the following protocol: 98°C for 1 min; 35 cycles of 98°C for 20s; 60°C for 15 s; 72°C for 35 s; 1 cycle 72°C for 15 min (Figure S1). Each step previously reported needed a purification step with SPRIselect beads to remove primer excess. Each sample was diluted to final concentration of 3.5 nM and was sequenced on a MiSeq (Figure S1).

**UMI-tagged Ig library analysis**

Figure S8 reports a schematic overview of steps performed in the analysis of UMI-tagged data. We designed UMI to resemble those previously published by Khan et. al. consisting in three degenerated 5 nt. portions interspaced by two spacers (Table S2) attached on both 5’ and 3’ ends.^20^ Spacers allowed to univocally identifying UMI regions removing possible small insertions/deletions. Moreover, non-G degenerated sequences allowed to partially identify substitutions occurring on UMIs to correct them, thus reducing the UMI numerosity. Lastly, applying UMI on both ends allowed accounting for different error rates in sequencing reads, thus performing read-specific error polishing. Similarly to what reported above, we designed a tailored workflow divided in two distinct parts: i) UMI_Analysis, to handle UMI-tagged reads and remove randomic errors and ii) UMI_Error-correction to remove systematic errors (Figure S8).

***i) UMI_Analysis***

After demultiplexing raw fastq files by bcl2fastq (v.2.6.1), read merging and read hard-filtering were performed with vsearch (v.2.14.2).^14^ Forward and reverse UMI (FWR_UMI, REV_UMI) were extracted with a custom python script and primers removed with cutadapt (v.2.3).^15^ Only reads having Ig primers cut were analyzed in the following steps. We performed an error correction procedure on FWR_UMIs and REV_UMIs which were clustered separately with a custom python script. The working principle was that ‘G’ nucleotides identified in degenerated portions were sequencing substitutions to cluster with UMIs non-containing ‘G’ with a density-based method (python sklearn package, DBSCAN method).^18^ UMI distances were calculated according to the Hamming distance with minor modification to handle ‘G’ as ‘N’ nucleotides. After the initial UMI correction, UMI were clustered with the UMIClusterer directional algorythm of umi_tools (v.1.0.1).^21^ Finally, clustered UMI-tagged reads were collapsed to generate consensus sequences for further analysis. We calculated the resultant base quality as the median Phred quality observed for each sequence belonging to specific clusters using the vsearch package. Sequences were collapsed with a python script and aligned with IgBlast against the IMGT reference database.^2,11^ Non-functional sequences were removed. ChangeO package (v.0.4.6) was exploited for clonotype assignment (see General pipeline for RepSeq analysis, Figure S3) and germline identification (Figure S8).^10^

***ii) UMI_Error-correction***

In principle, UMI were designed to remove randomic amplification errors generating during library preparation but not to eliminate high frequency systematic errors. To remove massive systematic sequencing errors we adapted principles of the error-polishing pipeline adopted for LymphoTrack data with slight modifications (see SSE correction pipeline, Figure S3). Consensus reads were re-aligned against the IMGT reference database,^2^ we then generated count matrices in a position and nucleotide-dependent way. For each position/nucleotide we calculated the median Phred quality weighted for the total number of nucleotides observed at specific positions, highlighting systematic errors characterized by median Phred scores <21. To correct erroneous positions we exploited the same decisional scheme adopted for LymphoTrack data (Figure S3 and S6). ID was calculated as reported above by means of iSI.

**Calculation of mutability in hotspot/coldspot**

CLL cells may undergo SHM due to the activity of Activation Induced Cytidine Deaminase (AID).^22–24^ A persistent activation of SHM enzymes could partially explain the ID observed in CLL.^25–28^ To demonstrate that ID is dependent on SHM machinery, we evaluated in silico whether mutations responsible for ID were compatible with known signatures. We employed igphyml (v.1.1.0), a phylogenetic algorythm designed to exploit the HLP19 substitution model to generate lineage trees taking into account SHM.^29,30^ The algorithm calculates variations in mutational load of specific nucleotidic motifs, namely WRC/GYW AID hotspot (known to be preferentially targeted by AID), and SYC/GRS AID coldspot (rarely targeted by AID mutational activity).^22–24^ We ran the algorythm with the following parameters: “ -t e --omega e,e --optimize lr “ on data corrected with our custom pipeline as described above. To further corroborate the results obtained, we calculated with a custom script the percentage of mutations compatible with SHM signatures per sample.

**References**

1. Leich E, Maier C, Bomben R, et al. Follicular lymphoma subgroups with and without t(14;18) differ in their N-glycosylation pattern and IGHV usage. *Blood Adv*. 2021;5(23):4890–4900.

2. Lefranc M-P, Giudicelli V, Duroux P, et al. IMGT®, the international ImMunoGeneTics information system® 25 years on. *Nucleic Acids Res*. 2015;43(Database issue):D413-22.

3. Rossi FM, Zucchetto A, Tissino E, et al. CD49d expression identifies a chronic-lymphocytic leukemia subset with high levels of mobilized circulating CD34+ hemopoietic progenitors cells. *Leukemia*. 2014;28(3):705–708.

4. D’Agaro T, Bittolo T, Bravin V, et al. NOTCH1 mutational status in chronic lymphocytic leukaemia: clinical relevance of subclonal mutations and mutation types. *Br J Haematol*. 2018;182(4):597–602.

5. Gattei V, Bulian P, Del Principe MI, et al. Relevance of CD49d protein expression as overall survival and progressive disease prognosticator in chronic lymphocytic leukemia. *Blood*. 2008;111(2):865–73.

6. Dal Bo M, Bulian P, Bomben R, et al. CD49d prevails over the novel recurrent mutations as independent prognosticator of overall survival in chronic lymphocytic leukemia. *Leukemia*. 2016;30(10):2011–2018.

7. Tissino E, Benedetti D, Herman SEM, et al. Functional and clinical relevance of VLA-4 (CD49d/CD29) in ibrutinib-treated chronic lymphocytic leukemia. *J Exp Med*. 2018;215(2):681–697.

8. Bomben R, Dal Bo M, Capello D, et al. Comprehensive characterization of IGHV3-21–expressing B-cell chronic lymphocytic leukemia: an Italian multicenter study. *Blood*. 2007;109(7):2989–2998.

9. Vander Heiden JA, Yaari G, Uduman M, et al. PRESTO: A toolkit for processing high-throughput sequencing raw reads of lymphocyte receptor repertoires. *Bioinformatics*. 2014;30(13):1930–1932.

10. Gupta NT, Vander Heiden JA, Uduman M, et al. Change-O: a toolkit for analyzing large-scale B cell immunoglobulin repertoire sequencing data. *Bioinformatics*. 2015;31(20):3356–3358.

11. Ye J, Ma N, Madden TL, Ostell JM. IgBLAST: an immunoglobulin variable domain sequence analysis tool. *Nucleic Acids Res*. 2013;41(Web Server issue):

12. Stern JNH, Yaari G, Vander Heiden JA, et al. B cells populating the multiple sclerosis brain mature in the draining cervical lymph nodes. *Sci Transl Med*. 2014;6(248):248ra107.

13. Chao A, Gotelli NJ, Hsieh TC, et al. Rarefaction and extrapolation with Hill numbers: a framework for sampling and estimation in species diversity studies. *Ecol Monogr*. 2014;84(1):45–67.

14. Rognes T, Flouri T, Nichols B, Quince C, Mahé F. VSEARCH: a versatile open source tool for metagenomics. *PeerJ*. 2016;4(10):e2584.

15. Martin M. Cutadapt removes adapter sequences from high-throughput sequencing reads. *EMBnet.journal*. 2011;17(1):10.

16. Schirmer M, Ijaz UZ, D’Amore R, et al. Insight into biases and sequencing errors for amplicon sequencing with the Illumina MiSeq platform. *Nucleic Acids Res*. 2015;43(6):e37.

17. Kozich JJ, Westcott SL, Baxter NT, Highlander SK, Schloss PD. Development of a dual-index sequencing strategy and curation pipeline for analyzing amplicon sequence data on the MiSeq Illumina sequencing platform. *Appl Environ Microbiol*. 2013;79(17):5112–20.

18. Ester M, Kriegel H-P, Sander J, Xu X. A Density-Based Algorithm for Discovering Clusters in Large Spatial Databases with Noise. 1996;

19. Schubert E, Sander J, Ester M, Kriegel HP, Xu X. DBSCAN Revisited, Revisited. *ACM Trans Database Syst*. 2017;42(3):1–21.

20. Khan TA, Friedensohn S, Gorter de Vries AR, et al. Accurate and predictive antibody repertoire profiling by molecular amplification fingerprinting. *Sci Adv*. 2016;2(3):e1501371.

21. Smith T, Heger A, Sudbery I. UMI-tools: modeling sequencing errors in Unique Molecular Identifiers to improve quantification accuracy. *Genome Res*. 2017;27(3):491–499.

22. Pilzecker B, Jacobs H. Mutating for Good: DNA Damage Responses During Somatic Hypermutation. *Front Immunol*. 2019;10:438.

23. Pettersen HS, Galashevskaya A, Doseth B, et al. AID expression in B-cell lymphomas causes accumulation of genomic uracil and a distinct AID mutational signature. *DNA Repair (Amst)*. 2015;25:60–71.

24. Maura F, Degasperi A, Nadeu F, et al. A practical guide for mutational signature analysis in hematological malignancies. *Nat Commun*. 2019;10(1):2969.

25. Gurrieri C, McGuire P, Zan H, et al. Chronic lymphocytic leukemia B cells can undergo somatic hypermutation and intraclonal immunoglobulin VHDJH gene diversification. *J Exp Med*. 2002;196(5):629–639.

26. Degan M, Bomben R, Bo MD, et al. Analysis of IgV gene mutations in B cell chronic lymphocytic leukaemia according to antigen-driven selection identifies subgroups with different prognosis and usage of the canonical somatic hypermutation machinery. *Br J Haematol*. 2004;126(1):29–42.

27. Bagnara D, Tang C, Brown JR, et al. Post-Transformation IGHV-IGHD-IGHJ Mutations in Chronic Lymphocytic Leukemia B Cells: Implications for Mutational Mechanisms and Impact on Clinical Course. *Front Oncol*. 2021;11(May):1–14.

28. Yuan C, Chu CC, Yan XJ, et al. The number of overlapping AID hotspots in Germline IGHV genes is inversely correlated with mutation frequency in chronic lymphocytic Leukemia. *PLoS One*. 2017;12(1):1–17.

29. Hoehn KB, Vander Heiden JA, Zhou JQ, et al. Repertoire-wide phylogenetic models of B cell molecular evolution reveal evolutionary signatures of aging and vaccination. *Proc Natl Acad Sci U S A*. 2019;116(45):22664–22672.

30. Hoehn KB, Lunter G, Pybus OG. A phylogenetic codon substitution model for antibody lineages. *Genetics*. 2017;206(1):417–427.

**Figure Legends**

**Figure S1. Library generation protocol for Immunoglobulin amplification with Unique Molecular Identifiers (UMI).** The scheme reports the steps adopted for the library generation of UMI-tagged immunoglobulins. i) RNA reverse transcription was performed with a JH-specific primer. ii) cDNA was amplified with a single-cycle PCR. iii) 30.000 molecules obtained by step ii were amplified with Illumina adapters.

**Figure S2. Flow-chart representation of the CLL samples analyzed in this study.** A) Samples are divided depending on the amplification protocol adopted (IGHV-Leader and FR1 Lymphotrack assays). Samples discarded due to the inability of identifying a prevalent pathological clone (Polyclonal) or with a total read number lower than 5000 are reported. B) Box plots show the number of sequences analyzed after each step of the pipeline, as reported in Figure S3, within the final cohort of 983 CLL patients.

**Figure S3. Bioinformatic pipeline for Lymphotrack RepSeq data.** The first block of the chart reports the generic bioinformatic pipeline adopted for canonical immunological repertoire (RepSeq) analysis. The second block of the chart reports the steps for the systematic sequencing errors (SSE) correction pipeline analysis performed on data generated with the generic pipeline for RepSeq data. The last block reports the steps for inverse Simpson Index calculation (iSI calculation). On the left side are depicted the single steps analysis, while on the right side are reported the packages used for specific steps.

**Figure S4. Read quality profile of Lymphotrack RepSeq data.** A) The heatmap reports the median Phred quality observed in MiSeq Read1 in dependence of I) the number of cycles, II) nucleotide observed, and III) preceding trinucleotidic motif. B) The heatmap reports the median Phred quality observed in MiSeq Read2 in dependence of I) the number of cycles, II) nucleotide observed, and III) preceding trinucleotidic motif. Phred score according to Illumina. C) The heatmap reports the median Phred quality observed for each base after the reconstruction of immunoglobulin in dependence of the IGHV families and the immunoglobulin position.

**Figure S5. Identification of systematic error based on median Phred quality.** The scatterplot depicts the percentage of single mutations observed by both FR2 and IGHV-Leader assays (blue), and by IGHV-Leader protocol only (red) in 62 CLL samples based on a frequency range of 0.1%-100% VAF. B) The boxplot reports the median Phred quality observed for the 1,349 mutations identified by IGHV-Leader assay only (orange box) and for the 1,672 mutations commonly identified by the IGHV leader and the IGHV FR2 assay.

**Figure S6. Schematic representation of the systematic sequencing errors (SSE) correction pipeline.** The scheme depicts the key steps adopted for the error correction. A) Example of cumulative frequency distribution in a position dependent manner of each nucleotide derived from realigned fastq of the specific CLL clone. In the reported example, at position 40 both A and T are represented in a discrete percentage of sequences (81% and 19%, respectively). B) Identification of possible errors. When multiple bases were called in the same position we calculated the base specific median Phred quality as reported by Illumina instrumentations. If the median quality Phred observed of each base is ≥21 both nucleotide were considered for analysis, otherwise the nucleotide with median quality Phred <21 was considered as a SSE and discarded (Italian Patent n.102022000027138_15422).

**Figure S7. Evaluation of the error-correction pipeline in IGHV-Leader and FR1 and comparison between Unique Molecular Identifier (UMI) and IGHV Leader protocol.** AB) Comparison between IGHV-Leader and FR1 comparison study. The scatterplots report the frequency of major (N=91, panel A) and minor (N= 1,013, panel B) subclones identified with both IGHV-Leader and FR1 assays with the systematic sequencing errors (SSE) correction pipeline. C) The scatterplot depicts the inverse Simpson index calculated for 91 samples amplified with both IGHV-Leader and FR1 assays and analyzed with the error-correction pipeline. Solid lines refer to the 1.2 iSi cut-off. DE) Comparison between UMI and IGHV Leader protocol. The scatter plot reports the frequency of the major (N=52, panel D) and minor (N=968, panel E) subclones identified with both UMI and IGHV Leader assays with the error-correction pipeline. F) The scatter plot depicts the inverse Simpson index (iSI) calculated for the 52 samples amplified with both UMI, and IGHV Leader and analyzed with the error-correction pipeline. Solid lines refer to the 1.2 iSi cut-off. Comparison between subclones derived from the different protocols was made comparing clones that have the exact same nucleotide sequences.

**Figure S8. Schematic representation of the bioinformatic pipeline for the analysis of RepSeq data tagged with Unique Molecular Identifiers (UMI).** The scheme reports the steps generally adopted for the analysis of RepSeq data with UMI. On the left side are depicted the single steps analysis, while on the right side are reported the packages used for specific steps.

**Figure S9. IGHV mutational status in the total cohort.** A) Kaplan-Meier curves comparing TTFT probabilities of 396 mutated (M) IGHV cases (green line), and 363 unmutated (U) IGHV cases (black line). The number of patients in each group is reported; P value refers to log-rank test.

**Figure S10. Pairwise comparisons between phylogenetic tree parameters.** Each set of boxplots represents a different graph metric. The y-axis represents the value of the metric, the horizontal line in the middle of the boxplot representing the median. P value refers to the overall Kruskal Wallis comparison. ** p value<0.01; *** p value<0.001; **** p value<0.0001; ns: not significant. On the right depicted an example of lineage tree and tree properties. Nodes in the tree can be either the root node (orange node), leaves (sequences of cells that had no descendants; green nodes), or internal nodes. Internal nodes can be either split nodes, those with more than one child (light blue nodes); or pass-through nodes, those with exactly one child (red nodes).

**Figure S11. Distribution of IGHV families and genes in the CLL cohort.** A) The barchart reports the number of CLL cases in dependence of IGHV families. B) The barchart reports the number of CLL cases in dependence of IGHV families divided according to the IGHV mutational status (mutated: M, unmutated: U). C) The barchart reports the number of samples with specific IGHV genes divided by mutational status.

**Figure S12. Top 10 IGHV genes with high intraclonal diversification (ID) among U and M-CLL.** The barchart reports the number of CLL cases for the top 10 IGHV with ID_high_ according to the IGHV mutational status (unmutated: U, mutated: M).

**Figure S13. Immunophenotypic analysis of cases with high intraclonal diversification (ID) in M-CLL.** The violin plots report the distribution of MFI values for each specific marker based on the presence or not of ID. Green plot represent mutated IGHV (M-CLL) and ID_low_ cases, purple plot represent M-CLL and ID_high_ cases.

**Figure S14. Variation in the number of mutations compatible with Activation Induced Cytidine Deaminase (AID) and polymerase eta.** The boxplots report the number of mutations compatible with AID mutational activity in both forward (WRC, W=A,T , R=A,G), and reverse strands (GYW, Y=C,T , W=A,T). The number of mutations occurring in AID coldspots for both the forward and reverse strand are reported in the middle (SYC and GRS, S=G,C ). The boxplots on the bottom report the number of mutations compatible with pol eta (η) in both forward, and reverse strands. The horizontal line in the middle of the boxplot represents the median. Analysis was performed by excluding all the shared mutations and circumscribing the analysis to the partially shared/unique mutations, i.e. the mutations allegedly acquired after the neoplastic transformation. P value refers to the overall Kruskal Wallis comparison.

**Figure S15. Variation in percentage of replacement and silent mutations in the FR and CDR region.** The boxplots report the percentage of S and R mutations calculated on 840 CLL samples. The horizontal line in the middle of the boxplot represents the median. *P* value refers to t-test.

**Figure S16. Clonal evolution of ID in longitudinal ID_high_ samples.** Line chart showing the iSI score calculated in 21 ID_high_ and 12 ID_low_ CLL patients with longitudinal samples.
