# Supplementary material for: Measurement of Immunoglobulin Intraclonal diversification refines the clinical impact of IGHV mutational status in chronic lymphocytic leukemia
Source: Leukemia. 2025 Jun 18;39(8):1905–14. doi: 10.1038/s41375-025-02650-2 (PMC12310548; doi:10.1038/s41375-025-02650-2)

Figure S1

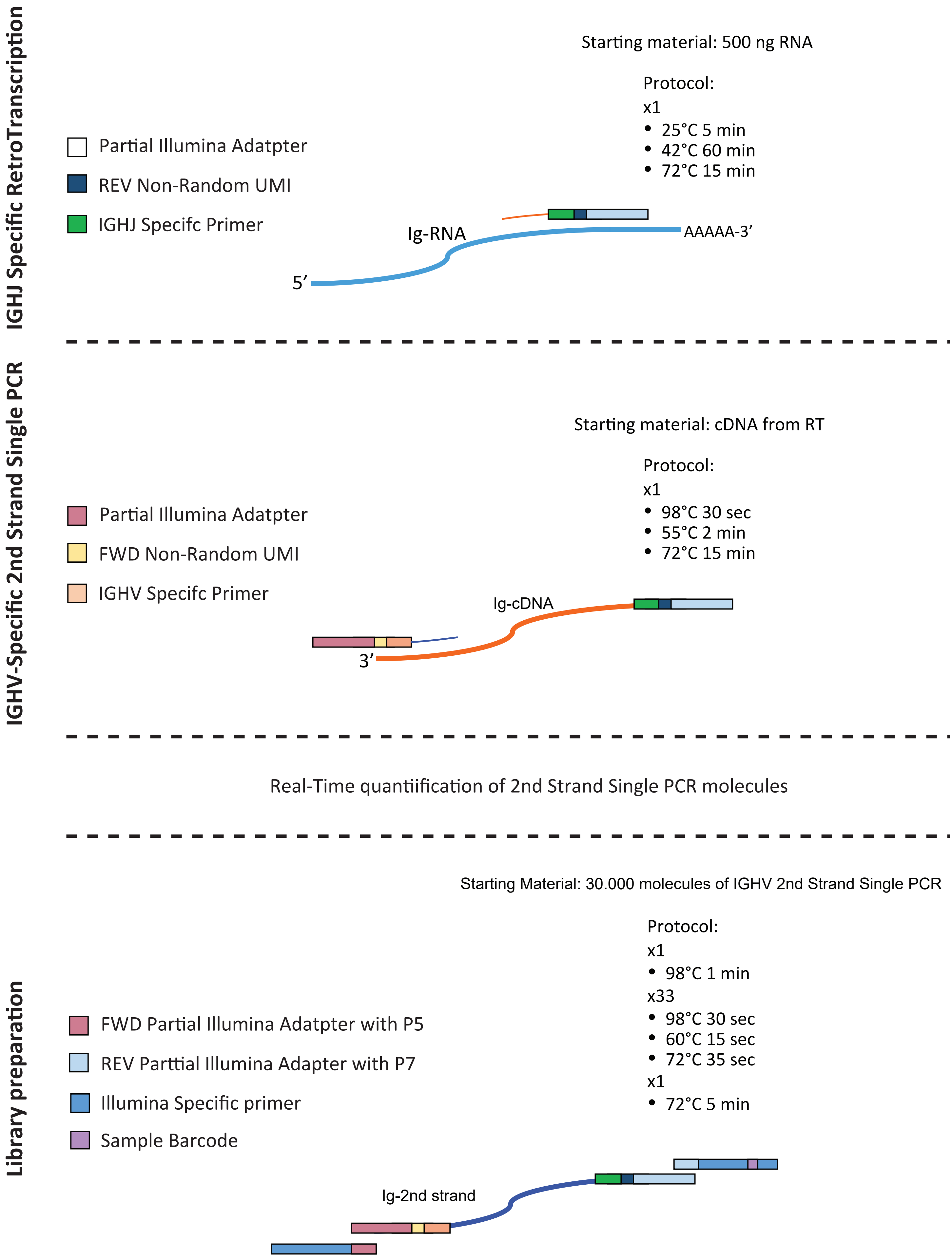
IGHJ Specific RetroTranscription
Partial Illumina Adatpter
REV Non-Random UMI
IGHJ Specifc Primer
Starting material: 500 ng RNA
Protocol:
x1
• 25°C 5 min
• 42°C 60 min
• 72°C 15 min
Ig-RNA
5'
AAAAA-3'
IGHV-Specific 2nd Strand Single PCR
Partial Illumina Adatpter
FWD Non-Random UMI
IGHV Specifc Primer
Starting material: cDNA from RT
Protocol:
x1
• 98°C 30 sec
• 55°C 2 min
• 72°C 15 min
Ig-cDNA
3'
Real-Time quantiification of 2nd Strand Single PCR molecules
Library preparation
Starting Material: 30.000 molecules of IGHV 2nd Strand Single PCR
Protocol:
x1
• 98°C 1 min
x33
• 98°C 30 sec
• 60°C 15 sec
• 72°C 35 sec
x1
• 72°C 5 min
FWD Partial Illumina Adatpter with P5
REV Parttial Illumina Adapter with P7
Illumina Specific primer
Sample Barcode
Ig-2nd strand

Figure S2

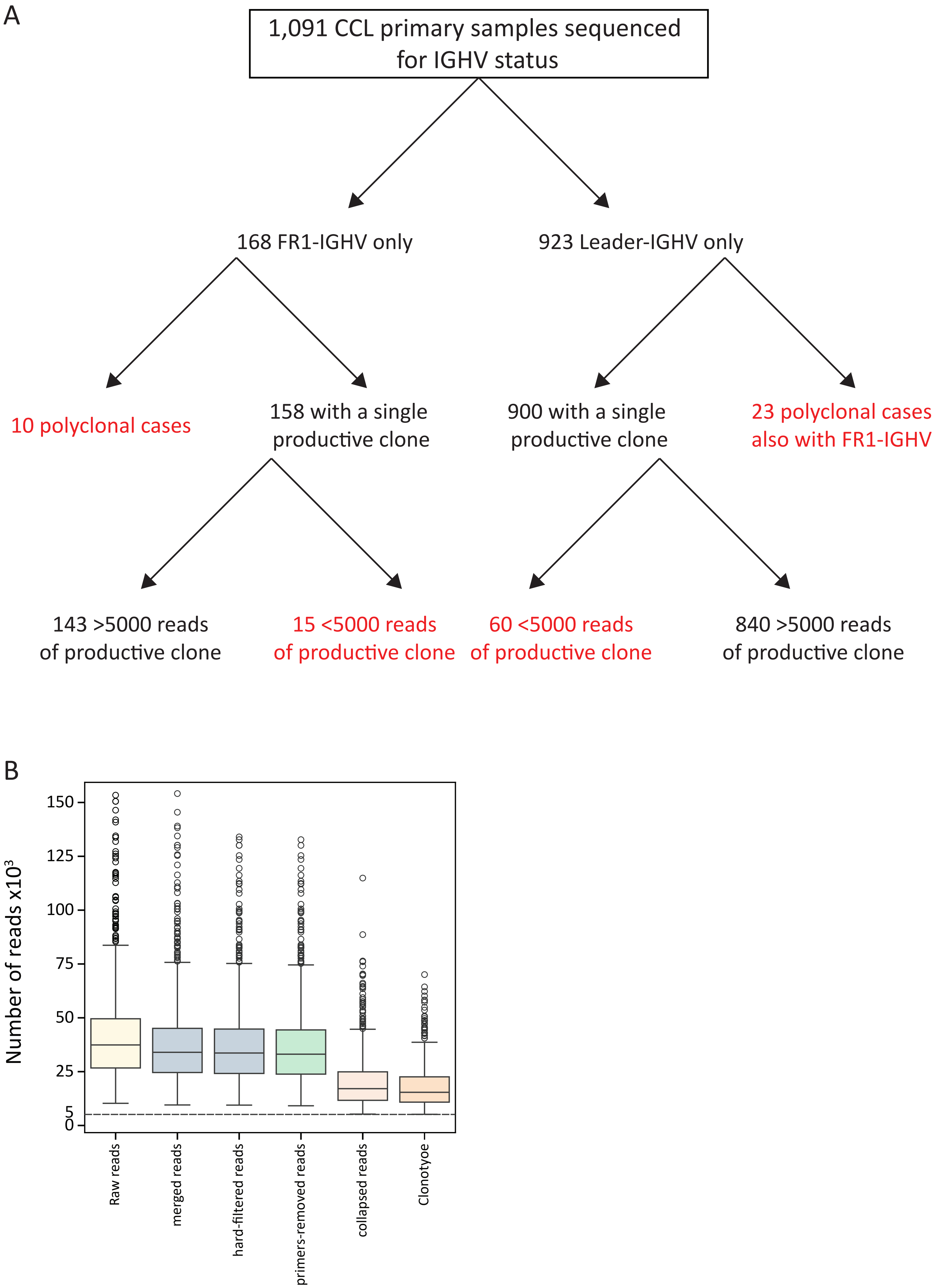
A
1,091 CCL primary samples sequenced for IGHV status
168 FR1-IGHV only
923 Leader-IGHV only
10 polyclonal cases
158 with a single productive clone
900 with a single productive clone
23 polyclonal cases also with FR1-IGHV
143 >5000 reads of productive clone
15 <5000 reads of productive clone
60 <5000 reads of productive clone
840 >5000 reads of productive clone
B
Number of reads x10^3
150
125
100
75
50
25
5
0
Raw reads
merged reads
hard-filtered reads
primers-removed reads
collapsed reads
Clonotyoe

Figure S3

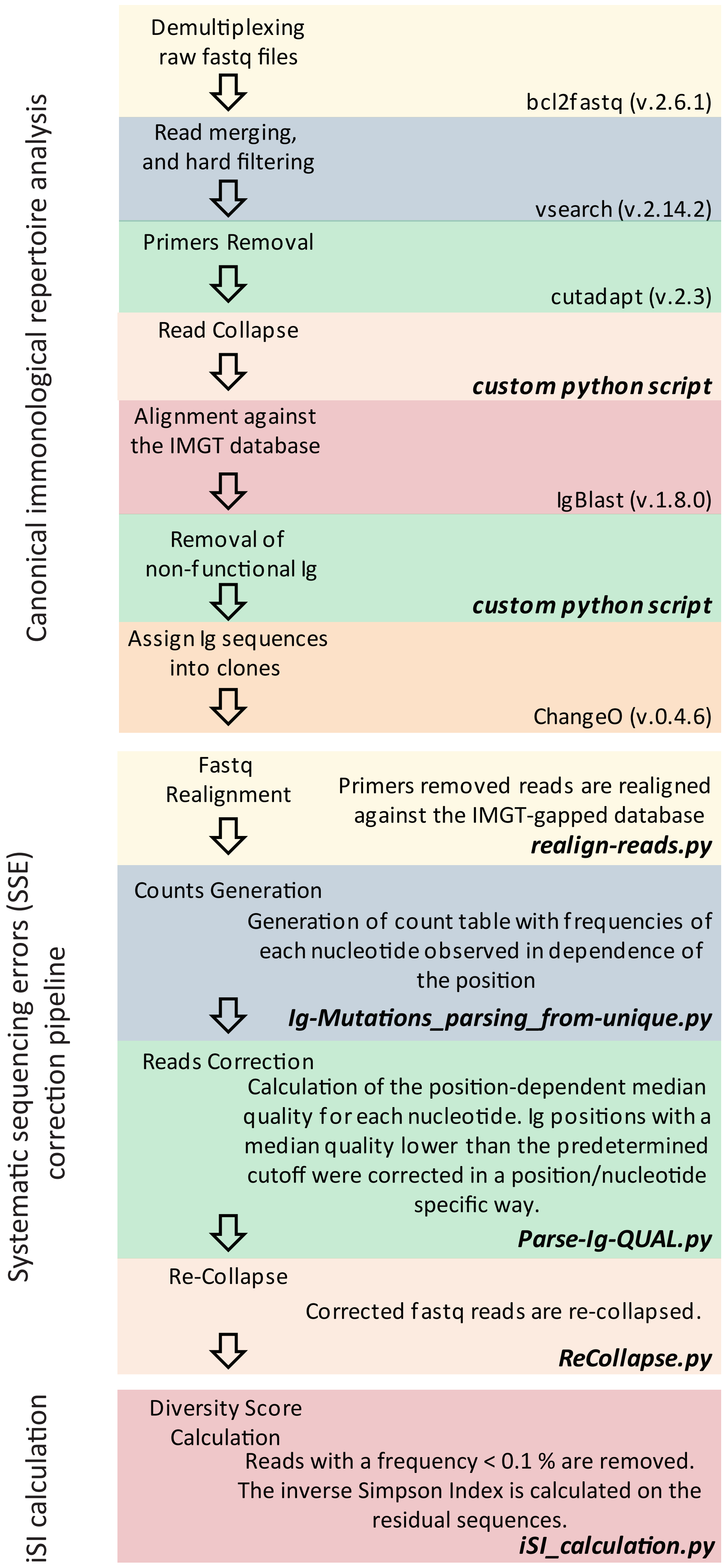

Canonical immonological repertoire analysis
Demultiplexing raw fastq files
bcl2fastq (v.2.6.1)
Read merging, and hard filtering
vsearch (v.2.14.2)
Primers Removal
cutadapt (v.2.3)
Read Collapse
custom python script
Alignment against the IMGT database
IgBlast (v.1.8.0)
Removal of non-functional Ig
custom python script
Assign Ig sequences into clones
ChangeO (v.0.4.6)
Systematic sequencing errors (SSE) correction pipeline
Fastq Realignment
Primers removed reads are realigned against the IMGT-gapped database
realign-reads.py
Counts Generation
Generation of count table with frequencies of each nucleotide observed in dependence of the position
Ig-Mutations_parsing_from-unique.py
Reads Correction
Calculation of the position-dependent median quality for each nucleotide. Ig positions with a median quality lower than the predetermined cutoff were corrected in a position/nucleotide specific way.
Parse-Ig-QUAL.py
Re-Collapse
Corrected fastq reads are re-collapsed.
ReCollapse.py
iSI calculation
Diversity Score Calculation
Reads with a frequency < 0.1 % are removed. The inverse Simpson Index is calculated on the residual sequences.
iSI_calculation.py

Figure S4

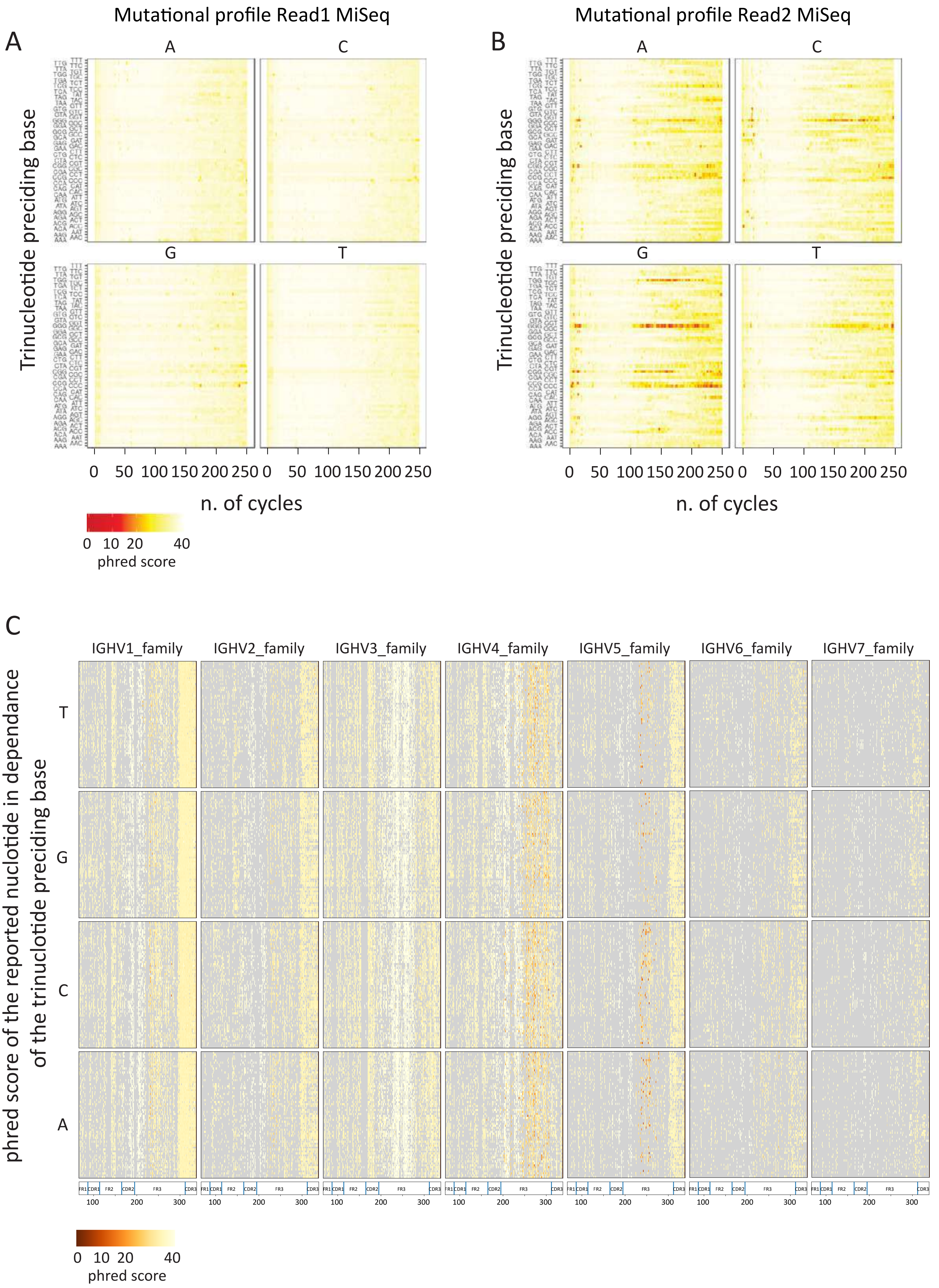

Mutational profile Read1 MiSeq
Mutational profile Read2 MiSeq
A
B
A
C
G
T
Trinucleotide preciding base
0 50 100 150 200 250
n. of cycles
0 10 20 40
phred score
C
IGHV1_family
IGHV2_family
IGHV3_family
IGHV4_family
IGHV5_family
IGHV6_family
IGHV7_family
T
G
C
A
phred score of the reported nuclotide in dependance of the trinuclotide preciding base
FR1 CDR1 FR2 CDR2 FR3 CDR3
100 200 300
0 10 20 40
phred score

Figure S5

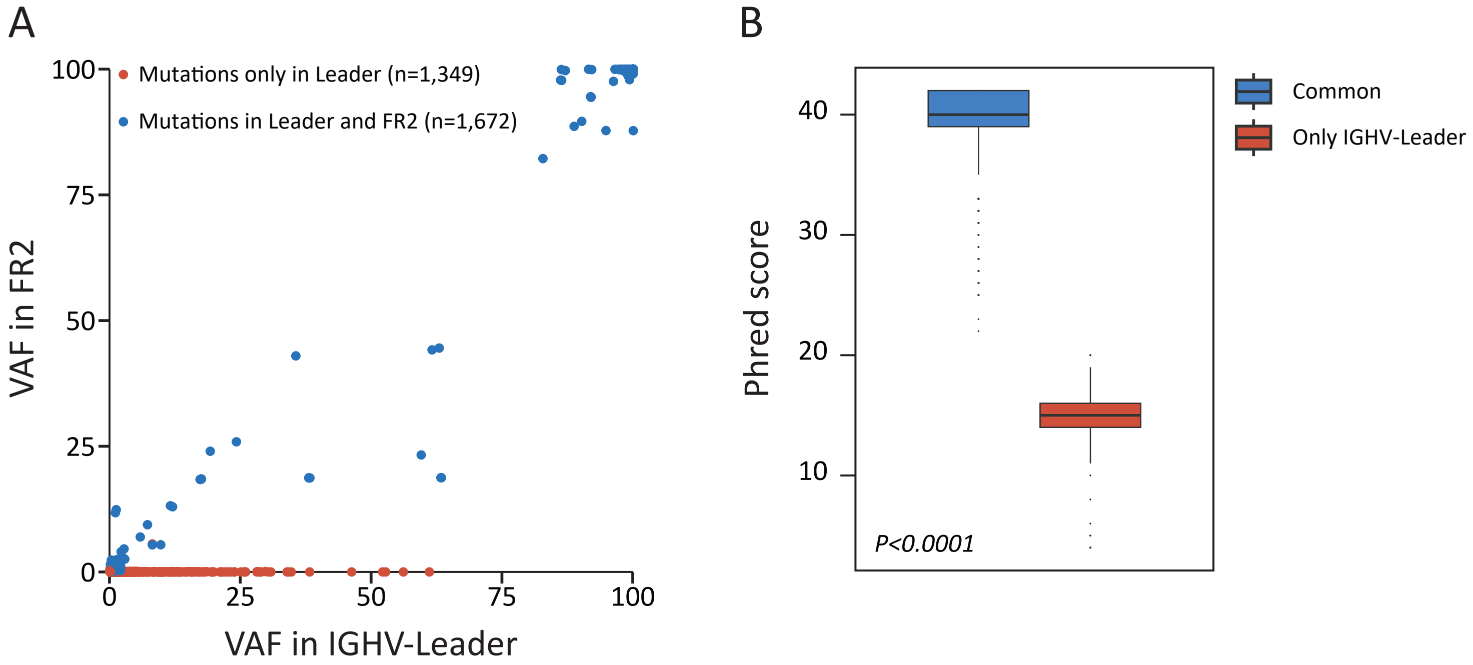
A
Mutations only in Leader (n=1,349)
Mutations in Leader and FR2 (n=1,672)
VAF in FR2
VAF in IGHV-Leader
0
25
50
75
100
B
Phred score
10
20
30
40
Common
Only IGHV-Leader
P<0.0001

Figure S6

A

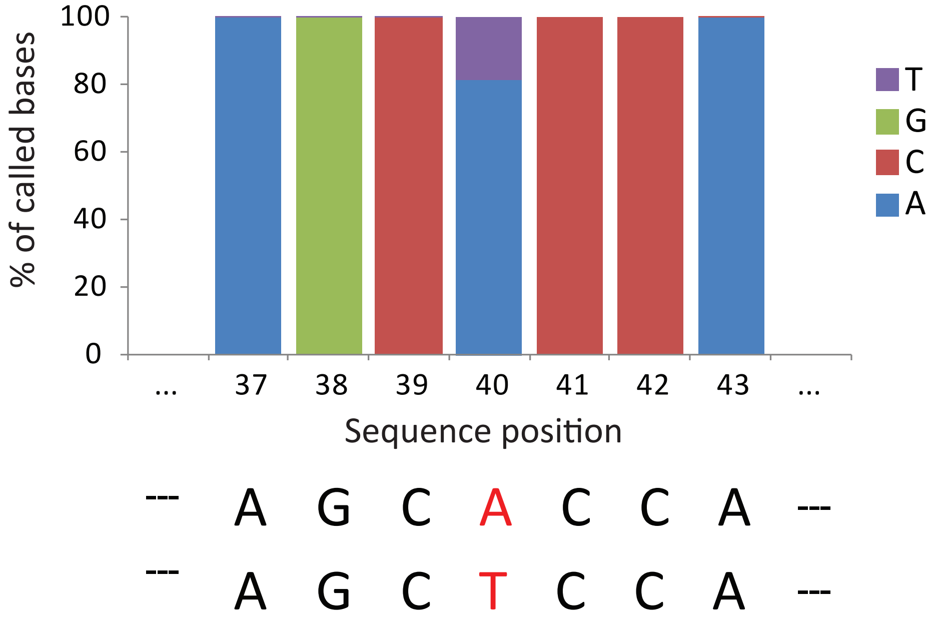
100
80
60
40
20
0
% of called bases
T
G
C
A
... 37 38 39 40 41 42 43 ...
Sequence position
--- A G C A C C A ---
--- A G C T C C A ---

B

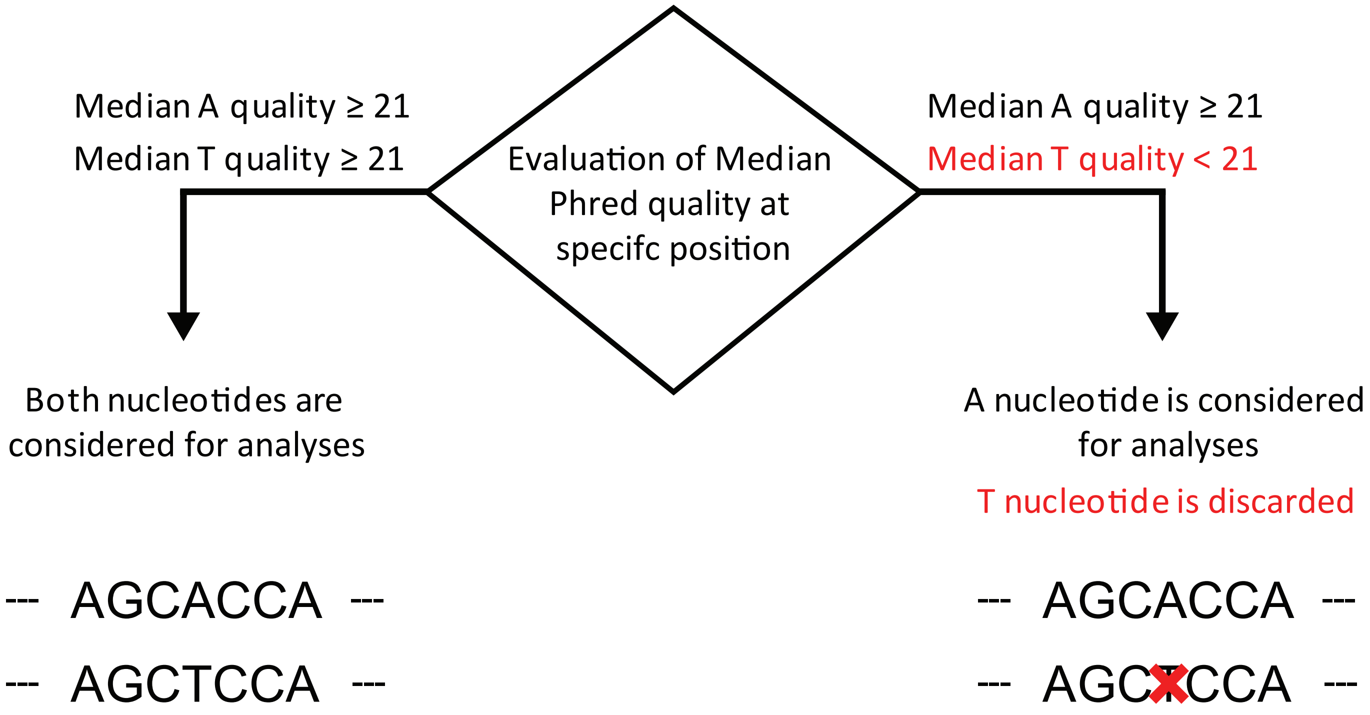
Median A quality ≥ 21
Median T quality ≥ 21
Evaluation of Median Phred quality at specifc position
Median A quality ≥ 21
Median T quality < 21
Both nucleotides are considered for analyses
A nucleotide is considered for analyses
T nucleotide is discarded
--- AGCACCA ---
--- AGCTCCA ---
--- AGCACCA ---
--- AGCTCCA ---

Figure S7

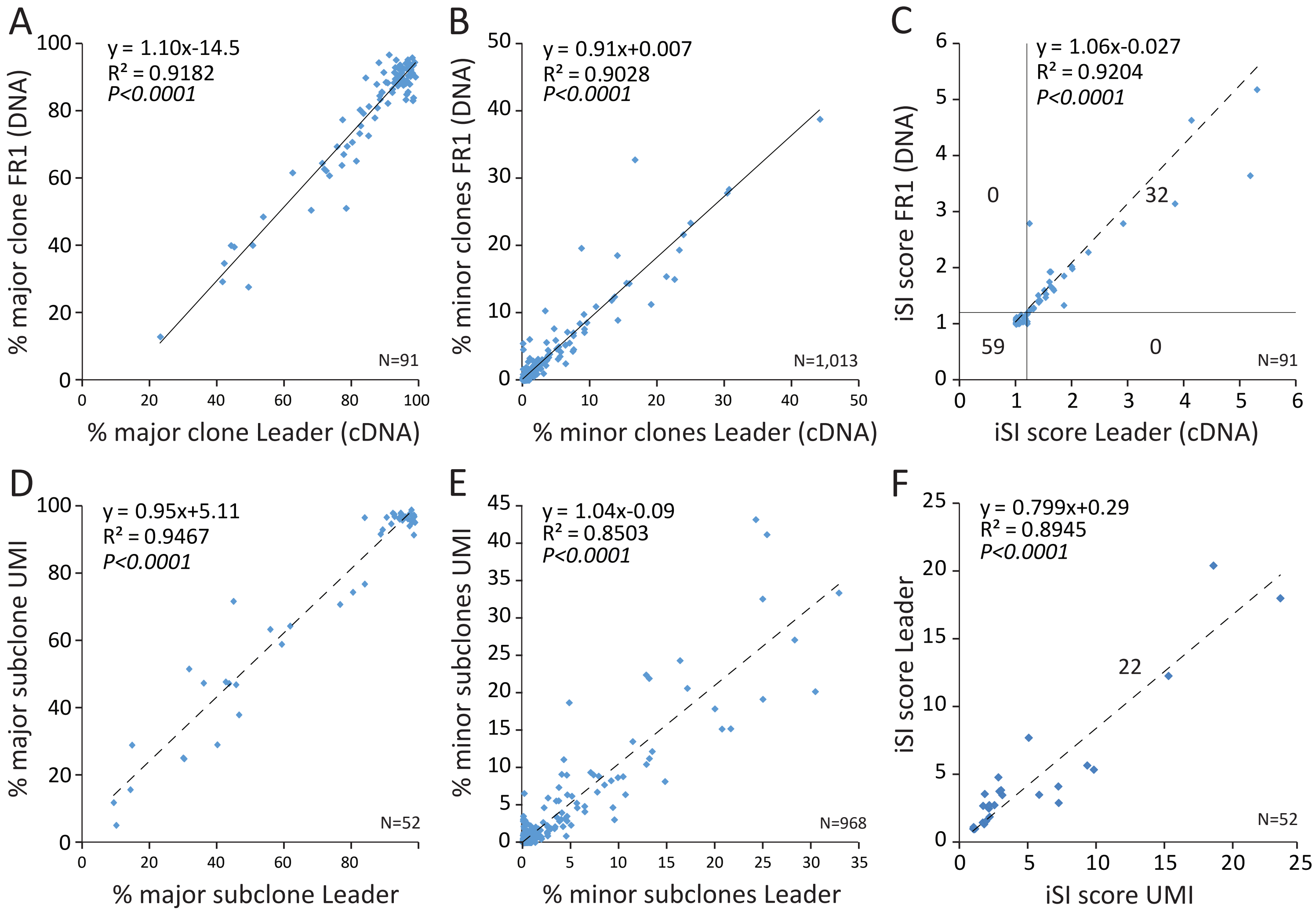

A
y = 1.10x-14.5
R^2 = 0.9182
P<0.0001
N=91
% major clone FR1 (DNA)
% major clone Leader (cDNA)
B
y = 0.91x+0.007
R^2 = 0.9028
P<0.0001
N=1,013
% minor clones FR1 (DNA)
% minor clones Leader (cDNA)
C
y = 1.06x-0.027
R^2 = 0.9204
P<0.0001
0
32
59
0
N=91
iSI score FR1 (DNA)
iSI score Leader (cDNA)
D
y = 0.95x+5.11
R^2 = 0.9467
P<0.0001
N=52
% major subclone UMI
% major subclone Leader
E
y = 1.04x-0.09
R^2 = 0.8503
P<0.0001
N=968
% minor subclones UMI
% minor subclones Leader
F
y = 0.799x+0.29
R^2 = 0.8945
P<0.0001
22
N=52
iSI score Leader
iSI score UMI

Figure S8

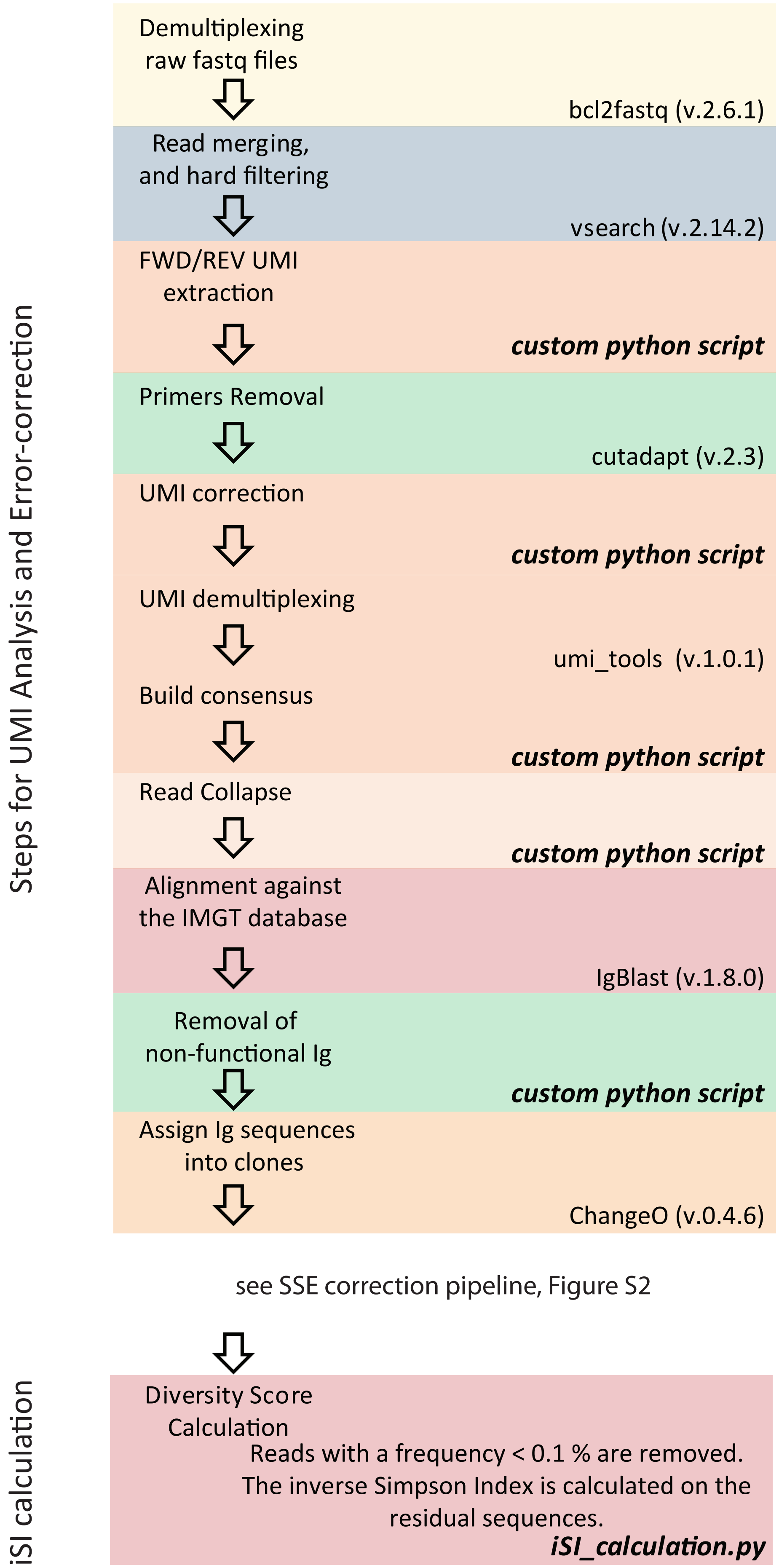
Steps for UMI Analysis and Error-correction
Demultiplexing raw fastq files
bcl2fastq (v.2.6.1)
Read merging, and hard filtering
vsearch (v.2.14.2)
FWD/REV UMI extraction
custom python script
Primers Removal
cutadapt (v.2.3)
UMI correction
custom python script
UMI demultiplexing
umi_tools (v.1.0.1)
Build consensus
custom python script
Read Collapse
custom python script
Alignment against the IMGT database
IgBlast (v.1.8.0)
Removal of non-functional Ig
custom python script
Assign Ig sequences into clones
ChangeO (v.0.4.6)
see SSE correction pipeline, Figure S2
iSI calculation
Diversity Score Calculation
Reads with a frequency < 0.1 % are removed.
The inverse Simpson Index is calculated on the residual sequences.
iSI_calculation.py

Figure S9

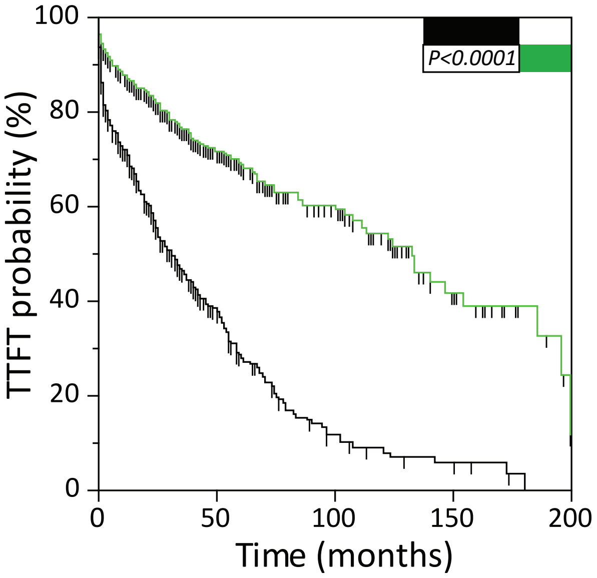

IGHV M	396	138	57	16	0
IGHV U	363	67	14	4	0

Figure S10

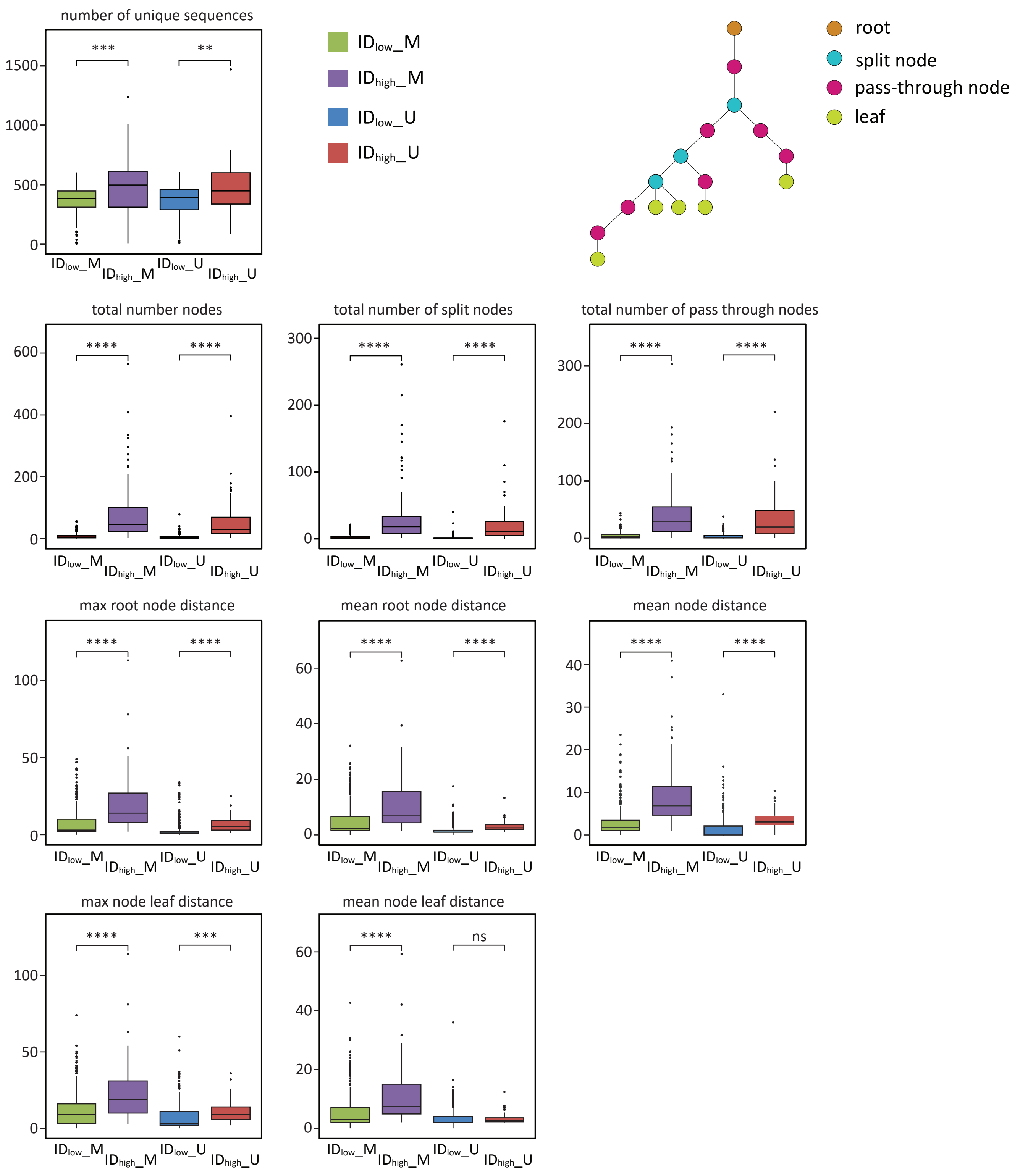

number of unique sequences
total number nodes
total number of split nodes
total number of pass through nodes
max root node distance
mean root node distance
mean node distance
max node leaf distance
mean node leaf distance
ID_{low}_M
ID_{high}_M
ID_{low}_U
ID_{high}_U
root
split node
pass-through node
leaf
****
***
**
ns

Figure S11

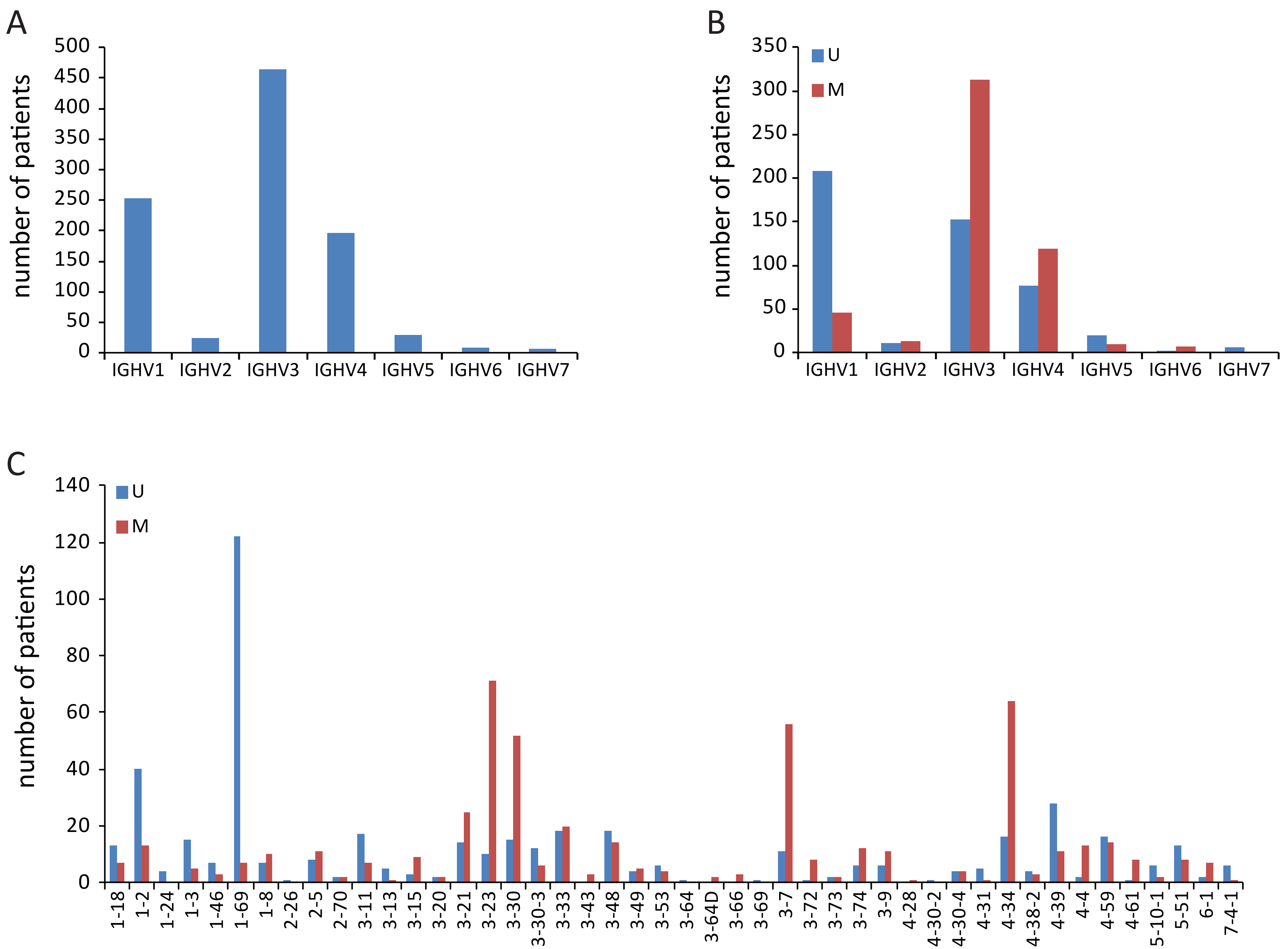
A
number of patients
500
450
400
350
300
250
200
150
100
50
0
IGHV1
IGHV2
IGHV3
IGHV4
IGHV5
IGHV6
IGHV7
B
U
M
number of patients
350
300
250
200
150
100
50
0
IGHV1
IGHV2
IGHV3
IGHV4
IGHV5
IGHV6
IGHV7
C
U
M
number of patients
140
120
100
80
60
40
20
0
1-18
1-2
1-24
1-3
1-46
1-69
1-8
2-26
2-5
2-70
3-11
3-13
3-15
3-20
3-21
3-23
3-30
3-30-3
3-33
3-43
3-48
3-49
3-53
3-64
3-64D
3-66
3-69
3-7
3-72
3-73
3-74
3-9
4-28
4-30-2
4-30-4
4-31
4-34
4-38-2
4-39
4-4
4-59
4-61
5-10-1
5-51
6-1
7-4-1

Figure S12

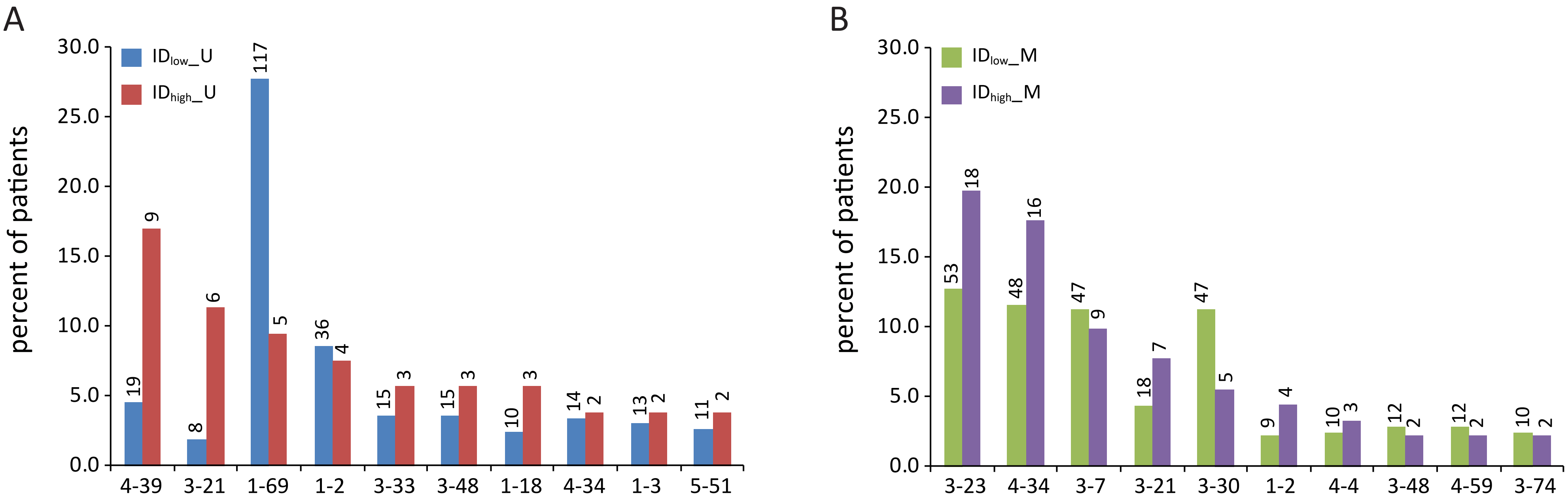
A
B
30.0
25.0
20.0
15.0
10.0
5.0
0.0
percent of patients
IDlow_U
IDhigh_U
19
9
8
6
117
5
36
4
15
3
15
3
10
3
14
2
13
2
11
2
4-39
3-21
1-69
1-2
3-33
3-48
1-18
4-34
1-3
5-51
IDlow_M
IDhigh_M
53
18
48
16
47
9
18
7
47
5
9
4
10
3
12
2
12
2
10
2
3-23
4-34
3-7
3-21
3-30
1-2
4-4
3-48
4-59
3-74

Figure S13

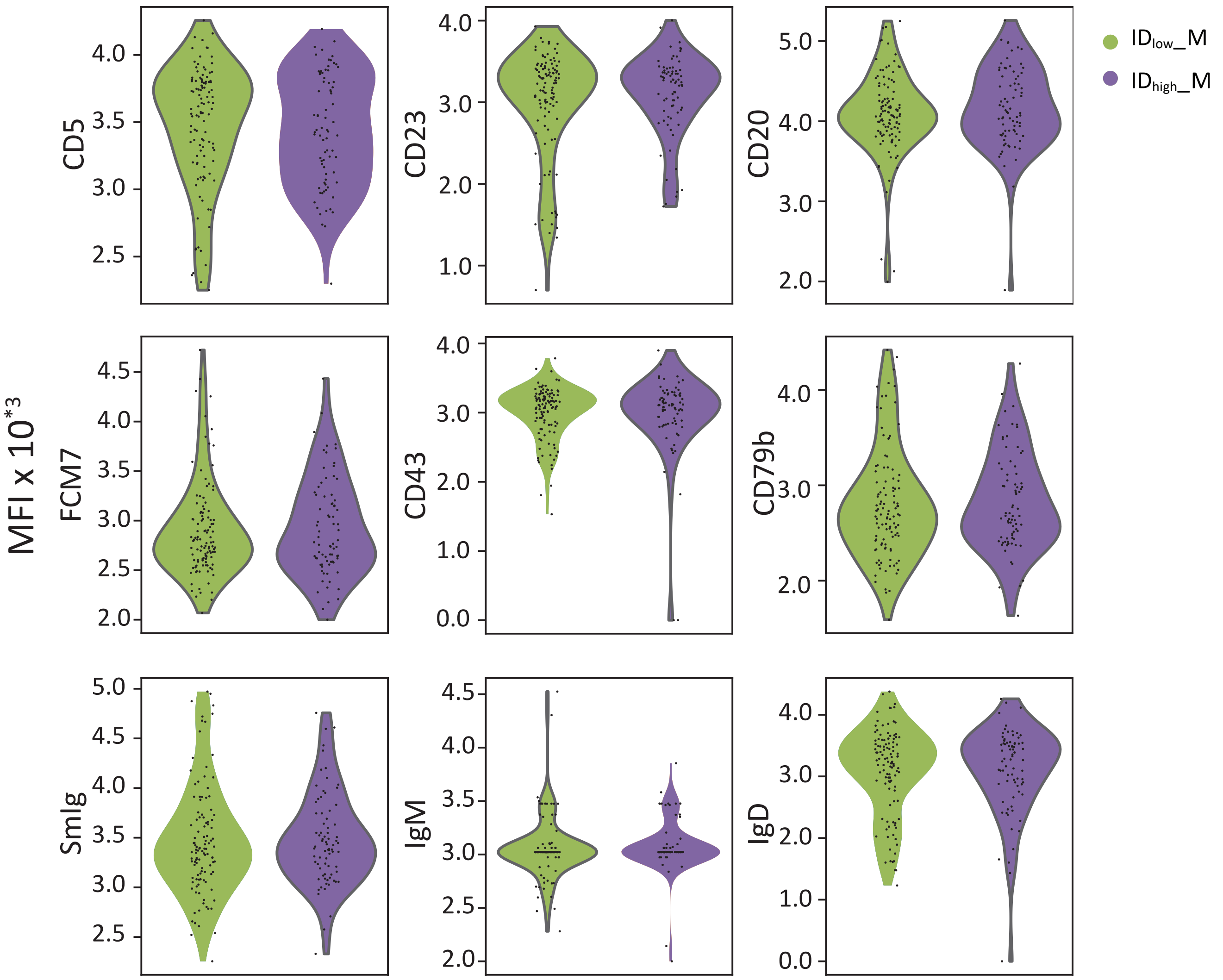

MFI x 10*3
CD5
CD23
CD20
FCM7
CD43
CD79b
SmIg
IgM
IgD
IDlow_M
IDhigh_M

Figure S14

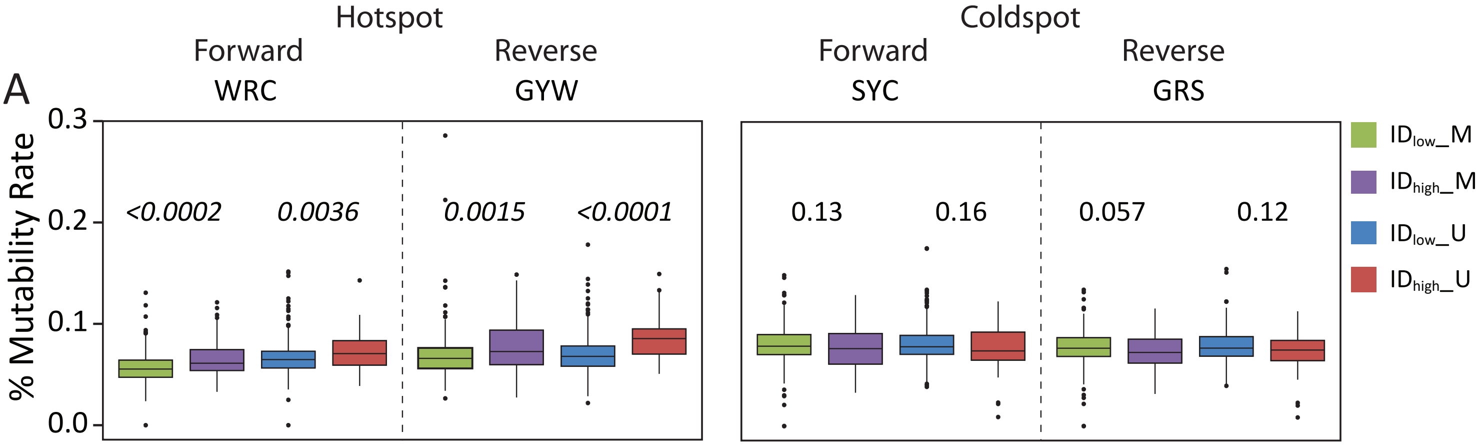
Hotspot
Forward
WRC
Reverse
GYW
Coldspot
Forward
SYC
Reverse
GRS
A
% Mutability Rate
0.3
0.2
0.1
0.0
<0.0002
0.0036
0.0015
<0.0001
0.13
0.16
0.057
0.12
ID_{low}_M
ID_{high}_M
ID_{low}_U
ID_{high}_U

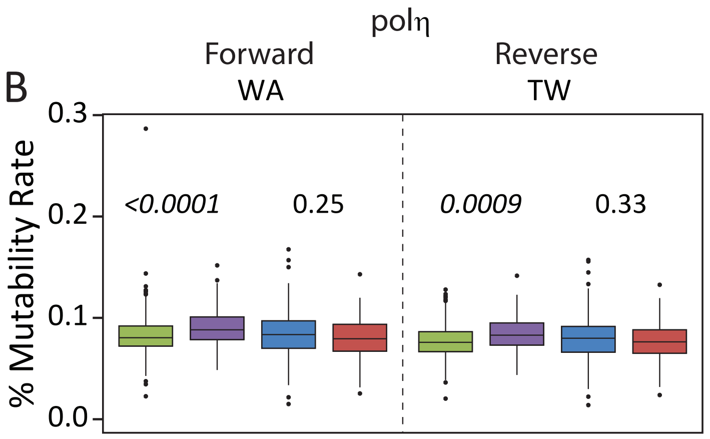
polη
Forward
WA
Reverse
TW
B
% Mutability Rate
0.3
0.2
0.1
0.0
<0.0001
0.25
0.0009
0.33

Figure S15

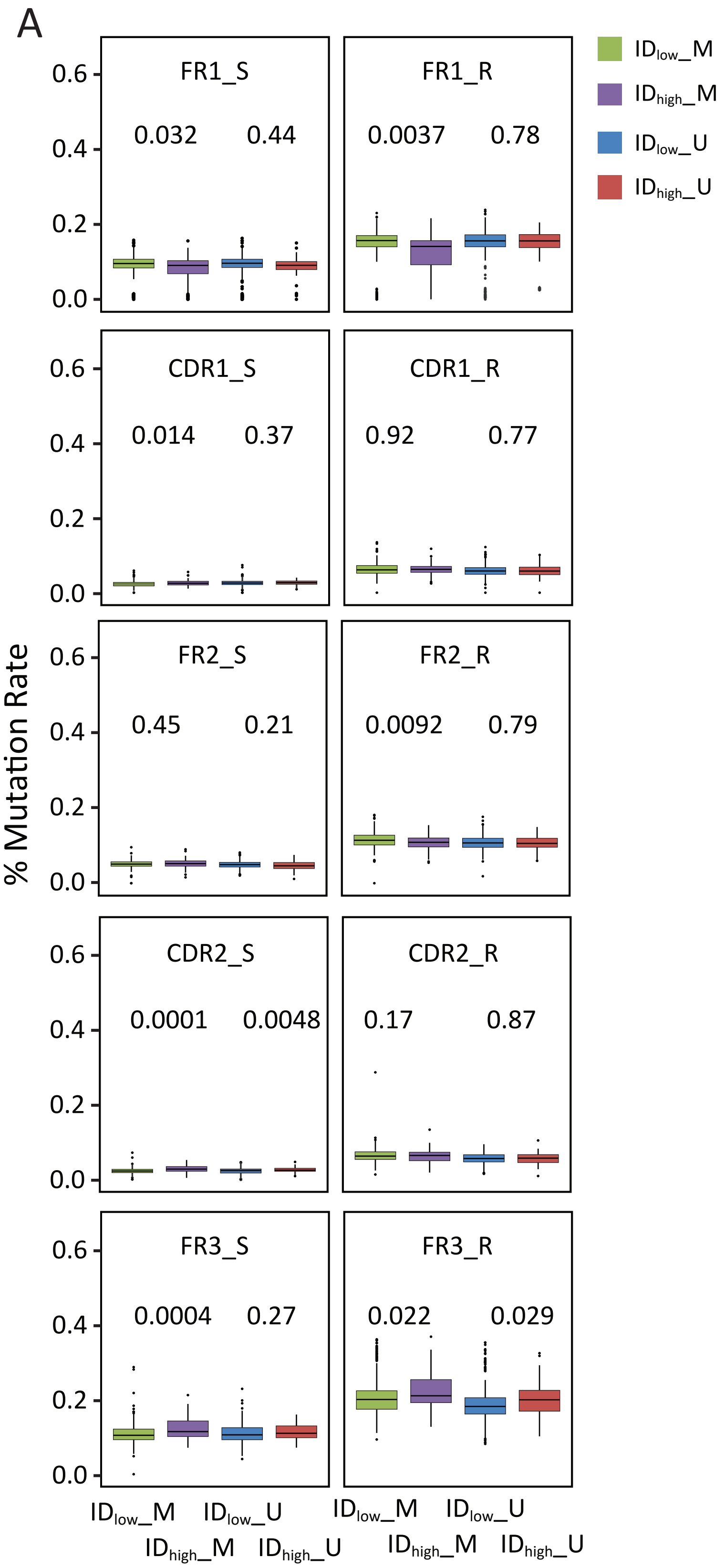
A
FR1_S
0.032
0.44
FR1_R
0.0037
0.78
CDR1_S
0.014
0.37
CDR1_R
0.92
0.77
FR2_S
0.45
0.21
FR2_R
0.0092
0.79
CDR2_S
0.0001
0.0048
CDR2_R
0.17
0.87
FR3_S
0.0004
0.27
FR3_R
0.022
0.029
% Mutation Rate
0.0
0.2
0.4
0.6
ID_low_M
ID_high_M
ID_low_U
ID_high_U

Figure S16

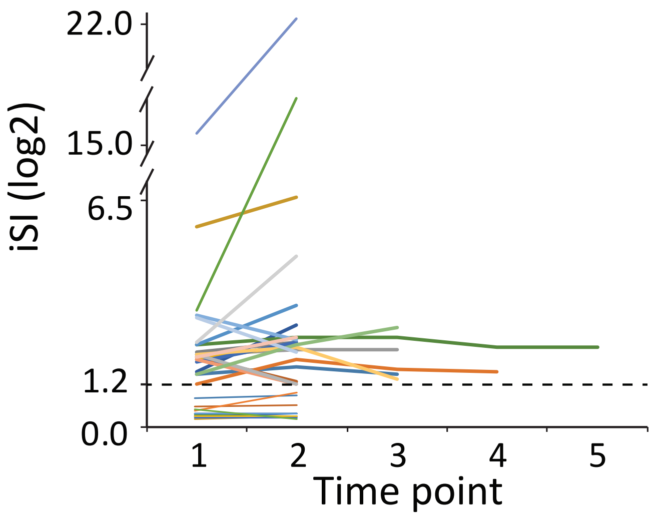

Supplement: Supplementary file 2 — Supplementary Figures [file 41375_2025_2650_MOESM2_ESM.pdf]
